# Template effects of vesicles in dynamic covalent chemistry†

**DOI:** 10.1039/d0sc03185b

**Published:** 2020-07-29

**Authors:** Carlo Bravin, Christopher A. Hunter

**Affiliations:** Department of Chemistry, University of Cambridge Lensfield Road Cambridge CB2 1EW UK herchelsmith.orgchem@ch.cam.ac.uk

## Abstract

Vesicle lipid bilayers have been employed as templates to modulate the product distribution in a dynamic covalent library of Michael adducts formed by mixing a Michael acceptor with thiols. In methanol solution, all possible Michael adducts were obtained in similar amounts. Addition of vesicles to the dynamic covalent library led to the formation of a single major product. The equilibrium constants for formation of the Michael adducts are similar for all of the thiols used in this experiment, and the effect of the vesicles on the composition of the library is attributed to the differential partitioning of the library members between the lipid bilayer and the aqueous solution. The results provide a quantitative approach for exploiting dynamic covalent chemistry within lipid bilayers.

## Abstract 2

Vesicle lipid bilayers have been employed as templates to modulate the product distribution in a dynamic covalent library of Michael adducts formed by mixing a Michael acceptor with thiols.

## Graphical abstract

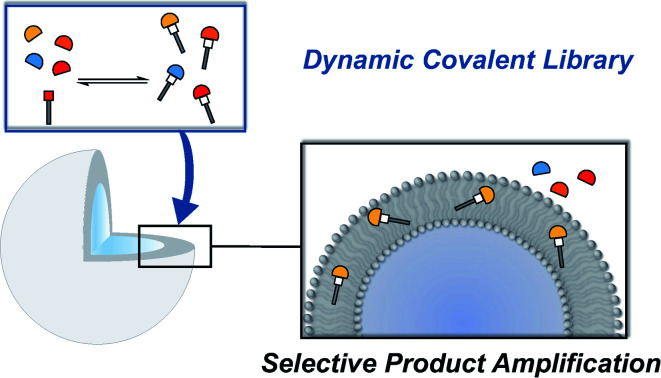

## Introduction

Dynamic covalent libraries (DCL) employ reversible covalent bonding to interchange different chemical components of compounds in a mixture.^1–5^ This synthetic tool has led to discoveries such as complex reaction networks,^6–8^ synthetic self-replicators,^9,10^ drug delivery systems^11–14^ and stimuli responsive assemblies.^15–19^ The composition of a DCL can be modulated by a template, which selectively amplifies a complementary molecular target in the mixture. A variety of template effects based on inorganic anions, organic molecules or external physical stimuli have been reported.^20^ DCLs have been studied in multiple solvents systems,^21,22^ at nanosystem interfaces,^23,24^ and in response to changes in solvent environment.^25,26^ Here we report the behaviour of a DCL in the multiphase environment provided by a membrane lipid bilayer in an aqueous phase, which opens the possibility for spatial and temporal control of the properties of the mixture.^27–30^

**Fig. 1:**
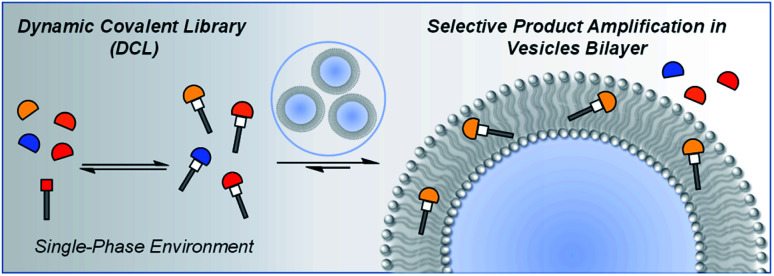
A vesicle can act as a template to change the composition of a dynamic covalent library.

Vesicles have been used to develop an understanding of molecular events at lipid bilayer interfaces in biological systems,^31–35^ synthetic membrane anchored receptors have been used for reaction control and transmembrane signal transduction,^36–41^ and a number of dynamic processes have been investigated in the presence of lipid bilayers.^42–44^ Here, we show that vesicles can be used as templates to modulate the product distribution of a DCL by exploiting the differential partitioning properties of the library components (Fig. 1).

## Results and discussions

**Scheme 1:**
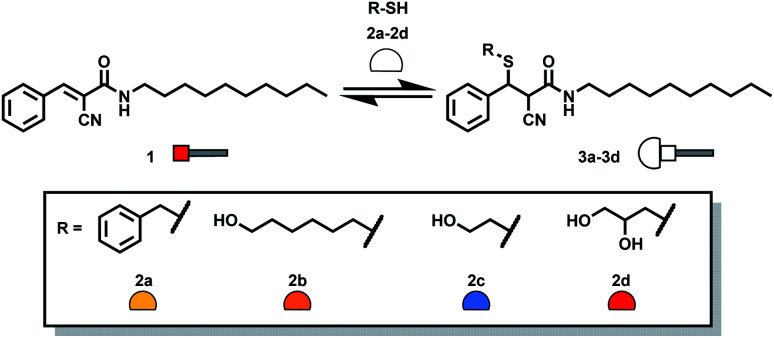
Addition of thiols **2a–2d** to **1** leads to formation of the corresponding Michael adducts **3a–3d**.

Michael acceptor **1** was obtained through a two-step synthesis involving formation of the cyanoacetamide of *n*-decylamine followed by an aldol condensation with benzaldehyde (Scheme 1, ESI Section S2†). The lipophilic chain of **1** is a membrane anchor, ensuring that the Michael acceptor is efficiently incorporated into vesicle membranes.

**Fig. 2:**
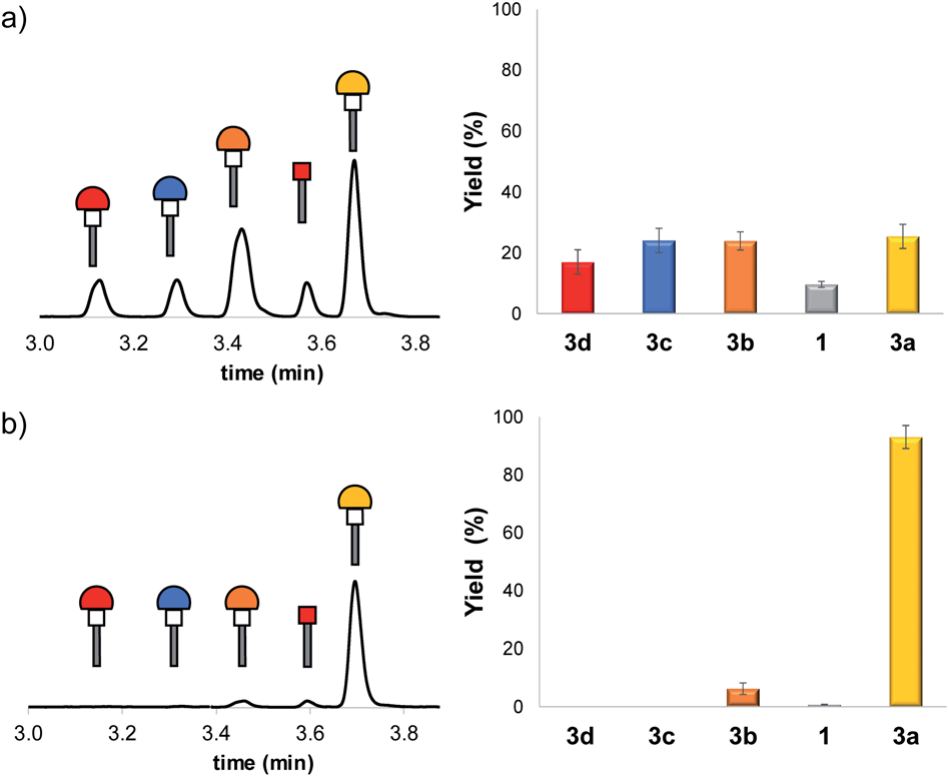
HPLC traces of the product distribution obtained 1 hour after mixing **1** (0.15 mM) with **2a–2e** (0.15 mM each) (a) in methanol, and (b) after addition of this mixture to an aqueous solution of vesicles (DOPC, 1 mM) containing 0.15 mM **1** in HEPES buffer at pH 7.2. The corresponding product distributions calculated from the integrals of the HPLC traces corrected by the relevant response factors are also shown.

Separate reaction of **1** with each of thiols **2a–2d** (Scheme 1) in methanol gave adducts **3a–3d** which could be characterised by HPLC-MS and HPLC-ELSD (ESI Section S4†). Mixtures of Michael acceptor **1** and thiols **2a–2d** were then used to form a DCL containing all of Michael adducts. Methanol was chosen for these control experiments to ensure good solubility of all library components and avoid any bias due to precipitation. A mixture containing each of the four thiols **2a–2d** at a concentration of 0.15 mM in methanol was prepared. Fig. 2a shows the HPLC trace obtained one hour after adding the thiol mixture to a 0.15 mM solution of **1**. A mixture of all four Michael adducts was obtained along with some unreacted **1**.

Fig. 2b shows the corresponding HPLC trace obtained one hour after adding the thiol mixture to an aqueous solution of DOPC vesicles containing 0.15 mM **1** in HEPES buffer at pH 7.2 (ESI Section S3†). There is a dramatic shift in the product distribution with formation of a single major product **3a** in the presence of vesicles. Addition of the same thiol mixture to an aqueous solution containing only HEPES buffer at pH 7.2 gave a mixture of all four Michael adducts, which demonstrates that the change in product distribution is due to the presence of the vesicles and is not due to phase separation or precipitation of some of the library components in water (ESI Section S5 and Fig. S16†). Experiments were carried out to demonstrate that the thiol–Michael addition reactions were occurring under reversible conditions. In separate experiments, four different libraries each composed of three of the thiols were equilibrated with **1** in methanol for one hour, then the fourth thiol was added, and the system was allowed to re-equilibrate. In each case, the same product distribution was obtained (ESI Section S4.3†), confirming the reversibility of the process.

**Fig. 3:**
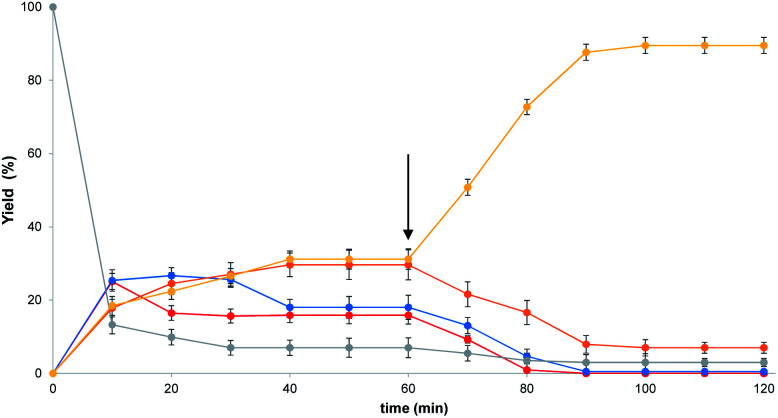
Time course for equilibration of the dynamic covalent library. At time zero, **1** (3 mM) was mixed with **2a–2e** (3 mM each) in 1.0 mL of methanol. After 1 hour (arrow), an aliquot of this mixture (0.1 mL) was added to 2.0 mL of a vesicle solution (1 mM DOPC) in HEPES buffer at pH 7.2. The fraction of each product was calculated from the integrals of the HPLC traces corrected by the relevant response factors. [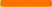] = **3a**, [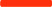] = **3b**, [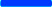] = **3c**, [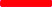] = **3d**, [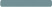] = **1**.

Fig. 3 shows the evolution with time of the DCL containing **1** and all four thiols. The mixture was fully equilibrated after one hour in methanol. At this point, vesicles were added and a change in the product distribution was observed (arrow in Fig. 3). The system was fully re-equilibrated after one hour giving Micheal adduct **3a** as the only major product. The dynamic nature of this process was demonstrated by equilibrating thiols **2b**, **2c** and **2d** with vesicles containing **1**, and then adding thiol **2a** after one hour. Michael adduct **3b** was initially observed as the major product. On addition of **2a**, rapid exchange occurred to give the same product distribution obtained by directly mixing all four thiols with vesicles containing **1**, with **3a** as the major product (ESI Fig. S14†).

**Fig. 4:**
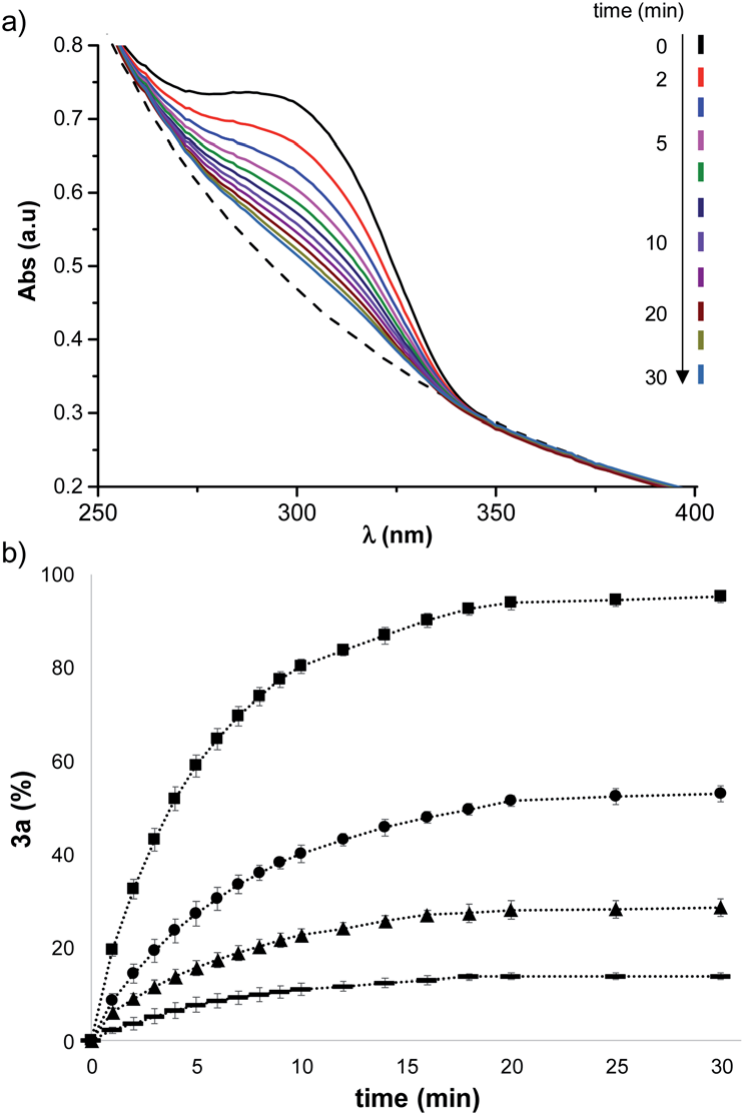
(a) UV-Vis absorption spectra recorded after addition of 1.0 eq. of **2a** to vesicles (1 mM DOPC) containing 0.15 mM **1** in HEPES buffer at pH 7.2. The black line is the spectrum at *t* = 0, and the dashed line is the spectrum of vesicles without **1**. (b) Time course for the formation of **3a** for addition of different amounts of **2a** to the solution of vesicles containing **1**: [**2a**] = 0.03 mM [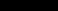], 0.06 mM [▲], 0.08 mM [●], 0.15 mM [■].

In order to understand the origin of the templating effect of vesicles in the DCL experiment, the reaction of each of the individual thiols with **1** was investigated. Michael acceptor **1** has a UV-Vis absorption band at 300 nm which disappears in the less conjugated Michael adduct.^45–47^ Even in the presence of vesicles, which cause some background scattering, the UV-Vis absorption band of **1** can be used to monitor reaction with a thiol. Fig. 4a shows UV-Vis absorption spectra recorded after addition of thiol **2a** to vesicles containing **1**. The formation of adduct **3a** was quantified by monitoring the disappearance of the absorption band at 300 nm, and Fig. 4b shows how the time course of the reaction depends on the concentration of **2a**.

The rate of formation of **3a** is similar in all of the experiments, and equilibration is complete after 30 minutes. However, the amount of **3a** formed depends strongly on the concentration of **2a**. The end points of the kinetics experiments in Fig. 4b were used to calculate an equilibrium constant of (2.1 ± 0.2) × 10^5^ M^−1^ for formation of the Michael adduct **3a** in the presence of vesicles. When the same experiment was carried out in methanol solution an equilibrium constant of (8.0 ± 0.7) × 10^2^ M^−1^ was obtained.

Equilibrium constants (*K*) for reaction with **1** were measured for each of the thiols, both in methanol solution and in the presence of vesicles. As expected from the DCL results shown in Fig. 2a, the equilibrium constants are practically identical in methanol (ESI Section S6.3†), indicating that there is no intrinsic difference in the reactivity of the thiols or the stability of the Michael adducts. In contrast in the presence of vesicles, the equilibrium constants span three orders of magnitude.

**Fig. 5:**
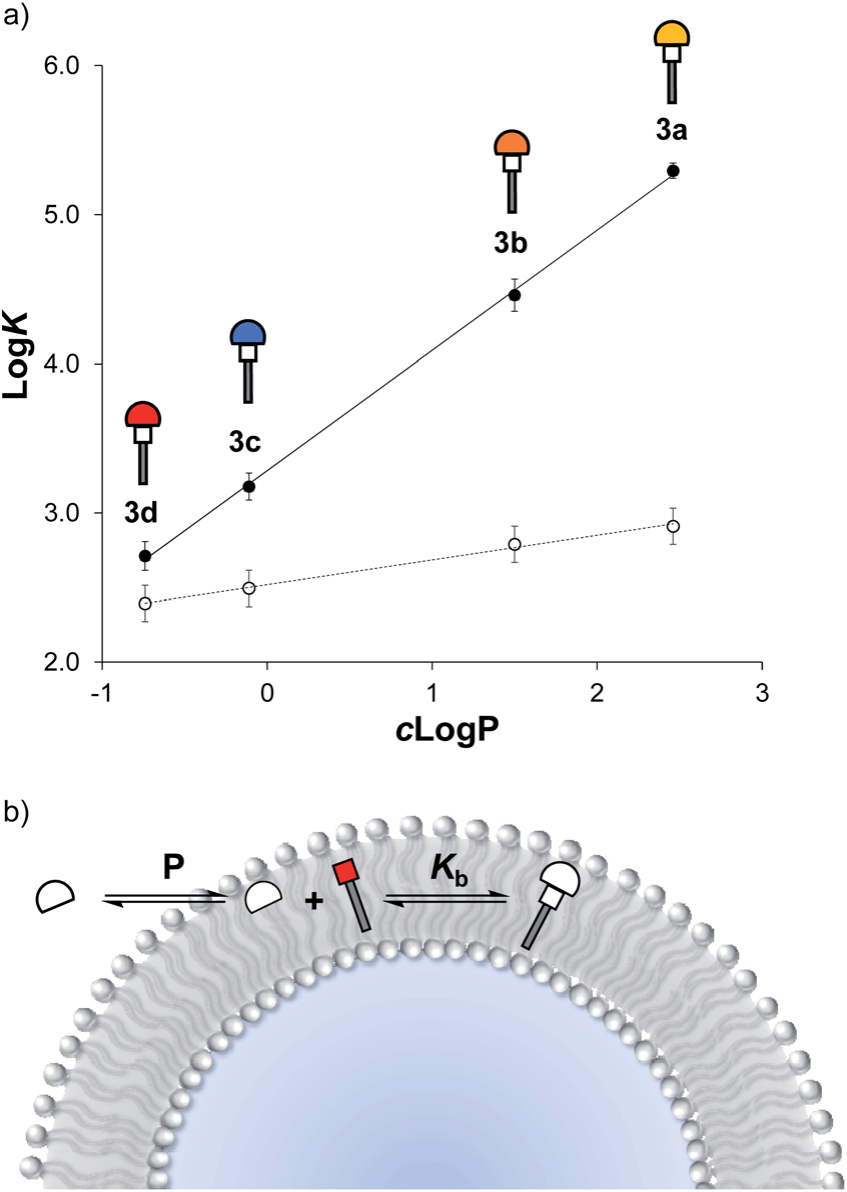
(a) Relationship between the equilibrium constant (log *K*) for formation of Michael adducts **3a–3d** in vesicles (●) or in methanol solution (○) and *c* log *P* of the corresponding thiols **2a–2e**. Straight lines of best fit are shown. (b) Coupled equilibria in formation of Michael adducts in vesicles.

Fig. 5a shows the relationship between the measured values of log *K* and the octanol–water partition coefficients of the corresponding thiols, *c* log *P*, calculated using MarvinSketch. The values of log *K* measured in vesicles correlate rather well with *c* log *P*, and the slope of the line of best fit is close to one, which suggests that the stability of the Michael adduct in the presence of vesicles is directly related to the hydrophobicity of the thiol.

An explanation for this result is shown in Fig. 5b. Reaction of a thiol with **1** in vesicles can be considered as two coupled equilibria: in the first step, the thiol partitions between the aqueous solution and the hydrophobic membrane with an equilibrium constant P, which depends on the solubility of the thiol; then the reaction with **1** takes place inside the lipid bilayer with an equilibrium constant *K*_b_, which is independent of the thiol.

## Conclusions

Vesicles have been used to template the product distribution in a DCL composed of a Michael acceptor and a mixture of thiols. In methanol solution, a mixture of all possible Michael adducts was observed, but addition of vesicles led to re-equilibration of the DCL to give a single major product. Equilibrium constants were measured for Michael adduct formation, and the results show that the origin of the template effect is differential partitioning of the reactants between the aqueous solution and hydrophobic membrane of the vesicles. These findings provide useful guidelines for the design of molecular components for exploiting dynamic covalent chemistry within lipid bilayers.

## Conflicts of interest

There are no conflicts to declare.

## Supplementary Material

SC-011-D0SC03185B-s001
