# Supplementary material for: Template effects of vesicles in dynamic covalent chemistry
Source: Chem Sci. 2020 Jul 29;11(34):9122–5. doi: 10.1039/d0sc03185b (PMC8163447; doi:10.1039/d0sc03185b)
Supplement: SC-011-D0SC03185B-s001 [file SC-011-D0SC03185B-s001.pdf]

Electronic Supplementary Material (ESI) for Chemical Science.
This journal is © The Royal Society of Chemistry 2020

Supporting Information for

Template Effects of Vesicles in Dynamic Covalent Chemistry

Authors: Carlo Bravin,[a] and Christopher A. Hunter[a*]

[a] Department of Chemistry, University of Cambridge, Lensfield Road, Cambridge CB2 1EW, U.K.

*Correspondence to: ch664@cam.ac.uk

Contents

1 General Methods ... 2
2 Synthesis and Characterization ... 3
2.1 Cyanoacetamide S1 ... 3
2.2 Receptor 1 ... 5
3 General procedure for vesicles preparation ... 7
4 Dynamic Covalent Libraries Analysis ... 8
4.1 LC-MS identification of 3a-3d Adducts ... 8
4.2 HPLC-ELSD Analysis of the Dynamic Covalent Library ... 11
4.3 Dynamic Covalent Library Reversibility Assessment ... 14
5 Controlled Product Amplification Triggered by Vesicles ... 17
6 Kinetic Experiments and Binding Constant Determination ... 19
6.1 UV-Vis spectra of thiol addition in vesicles ... 19
6.2 UV-Vis spectra of thiol addition in methanol ... 20
6.3 Kinetic profile of the adduct formation and binding constant determination ... 21
References ... 28

1 General Methods

^{1}H NMR and ^{13}C NMR spectra were recorded on a 400-MHz Bruker spectrometer. Chemical shifts are reported as δ values in ppm. All the ^{1}H NMR spectra were referenced to residual isotopic impurity of $CDCl_3$ (7.26 ppm), or CD_3CN (1.98 ppm). ^{13}C NMR spectra (50 MHz) were referenced to the $CDCl_3$ peak (77.0 ppm). The following abbreviations are used in reporting the multiplicity for NMR resonances: s=single, d=doublet, t= triplet, and m=multiplet. The NMR data were processed using MestReNova 10.0.2.

High resolution electrospray ionization mass spectrometry (HRMS-ESI) was performed on Waters LCT Premier TOF Spectrometer or by the Mass Spectrometry Service at the Department of Chemistry. The LCMS analysis of samples was performed using Waters Acquity H-class UPLC coupled with a single quadrupole Waters SQD2. ACQUITY UPLC BEH C8 Column, 130Å, 1.7 µm, 2.1 mm X 50 mm was used as the UPLC column. The conditions of the UPLC method are as follows: Solvent A: Water +0.1% Formic acid; Solvent B: Acetonitrile +0.1% Formic acid; Gradient of 0-4 minutes 5% - 100%B + 1 minute 100% B with re-equilibration time of 2 minutes. Flow rate: 0.4 ml/min; Column temperature of 40 °C; Injection volume of 2 µL for DCL experiment in methanol, 2 µL for DCL experiment in vesicles. The signal was monitored at 254 nm. HPLC-ELSD analysis were performed using an Analytical HPLC Agilent HP1100. ACQUITY UPLC BEH C8 Column, 130Å, 2.5 µm, 6 mm X 75 mm was used as column. The conditions were as follows: Solvent A: Water +0.1% trifluoroacetic acid; Solvent B: Acetonitrile +0.1% trifluoroacetic acid; 1 minute 50%A + 50%B, Gradient of 1 minutes 50% - 100%B + 5 minute 100% B with re-equilibration time of 2 minutes. Flow rate: 1.0 ml/min; Injection volume of 2 µL for the methanol solution, 20 µL for vesicles. ELSD detector was set at Evaporator Temperature: 45°C. Nebulizer Temperature: 30° C.

Melting point measurements were performed on Mettler Toledo MP90. Infrared (IR) spectra were recorded on Bruker Alpha. FTIR Spectrometer with single reflection diamond Platinum ATR. UV-Vis spectra were recorded using a Cary 60 (Agilent Technologies) in Hellma Analytics Suprasil quartz cuvettes. pH measurements were conducted using a Mettler-Toledo "Seven Compact" pH meter equipped with an "In-lab Micro" electrode. GPC purification of vesicles was carried out using GE Healthcare PD-10 desalting columns prepacked with Sephadex G-25 medium.

Vesicles were prepared using Avestin "LiposoFast" extruder apparatus, equipped with polycarbonate membranes with 200 nm pores. Lipids were purchased from Sigma Aldrich and used without further purification. All the reagents and solvents were purchased from Sigma Aldrich and Acros and used without further purification.

2 Synthesis and Characterization

2.1 Cyanoacetamide S1

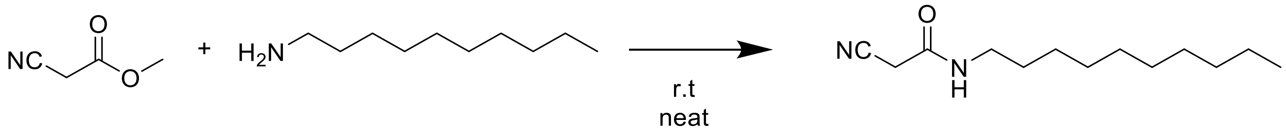

S1

Methyl cyanoacetate (0.79 ml, 9.0 mmol) and n-decylamine (1.54 ml, 7.5 mmol) were added together into a round bottom flask and stirred at room temperature. After 20 min, the precipitate was filtered and washed with cold diethyl ether then dried under vacuum. 25 ml dichloromethane were added, and the mixture was washed with 1N HCl, saturated bicarbonate and brine. The cyanoacetamide **S1** product was obtained as a white solid 1.309 g (78 %).

m.p. 82.5 °C

^{1}H NMR (400 MHz, $CDCl_3$) δ (ppm): 6.13 (s, 1H), 3.36 (s, 2H), 3.28 (m, 2H, J_1 = 8.0 Hz, J_2 = 4.0 Hz), 1.54 (m, 2H, J_1 = 8.0 Hz, J_2 = 4.0 Hz), 1.26 (m, 14H), 0.88 (t, 3H, J = 8.0 Hz).

^{13}C NMR (100 MHz, $CDCl_3$) δ (ppm): 160.67, 114.89, 40.50, 31.88, 29.52, 29.49, 29.29, 29.25, 29.21, 26.79, 25.84, 22.68.

HRMS (ESI-TOF) (*m/z*): $[M+H]^+$ calcd. for $[C_{13}H_{24}N_2O+H]^+$, 225.1889; found 225.1965.

FT IR (KBr) ν(cm^{-1}): 3296, 2915, 2849, 2240, 1650, 1554, 1338, 1228, 746, 717.

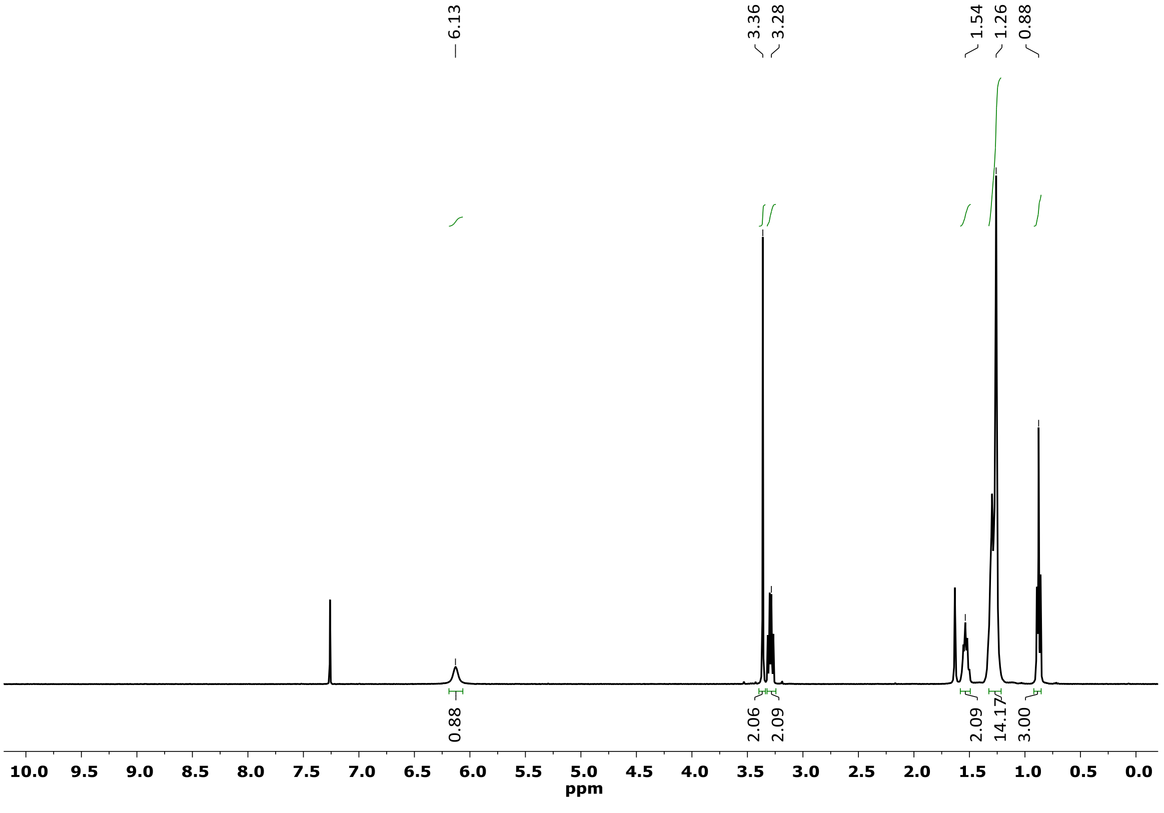

Figure S1 ^{1}H NMR (400 MHz, 301 K, $CDCl_3$) of cyanoacetamide **S1**.

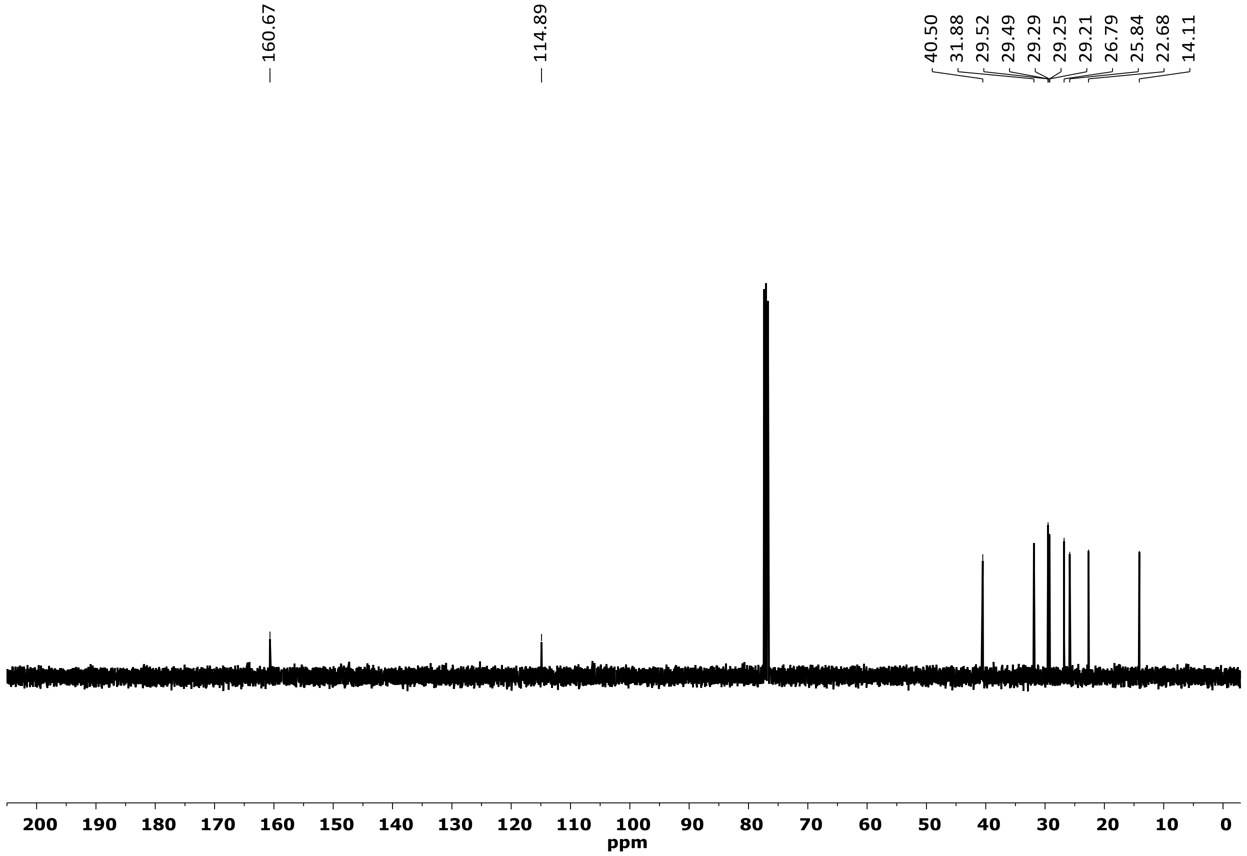

Figure S2 ^{13}C NMR (100 MHz, 301 K, $CDCl_3$) of cyanoacetamide **S1**.

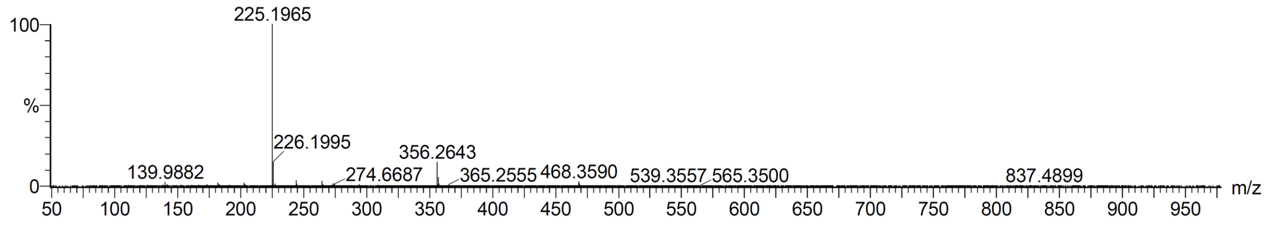

Figure S3 HRMS spectrum of (ESI-TOF) (m/z) of **S1**.

2.2 Receptor 1

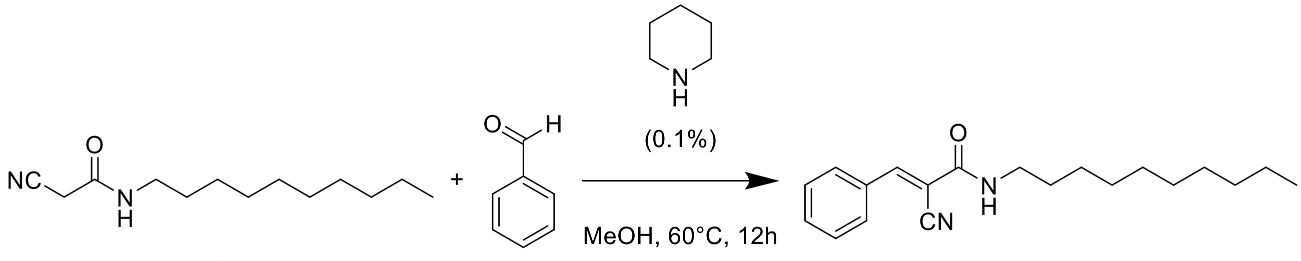

The cyanoacetamide **S1** (150 mg, 0.67 mmol) and benzaldehyde (68 μL, 0.67 mmol) were added together to 15 mL ethanol solution in the presence of catalytic amount of piperidine. The reaction mixture was stirred for 12 h at 60°C, and the solvent was evaporated. The residue was purified by column chromatography on silica gel (CH_2Cl_2:P.Et. 1:1) to give **1** (159 mg, 75%) as a white solid.

m.p. 71.2 ° C

^{1}H NMR (400 MHz, CD_3CN) δ (ppm): 8.18 (s, 1H), 7.93 (m, 2H, *J* = 8.0 Hz), 7.56 (m, 3H), 6.91 (s, 1H), 3.32 (m, 2H), 1.56 (m, 2H), 1.32 (m, 14H), 0.88 (t, 3H, *J* = 8.0 Hz).

^{13}C NMR (100 MHz, CD_3CN) δ (ppm): 160.41, 151.10, 132.28, 130.16, 129.15, 106.10, 39.95, 31.63, 29.26, 29.04, 28.97, 26.53, 22.38, 13.38.

HRMS (ESI-TOF) (*m/z*): $[M+H]^+$ calcd. for $[C_{20}H_{28}N_2O+H]^+$ 313.2202; found 313.2278

FT IR (KBr) ν(cm^{-1}): 3351, 3150, 2916, 2949, 2218, 1666, 1526, 1427, 1366, 1259, 1203, 745, 688, 598, 479,

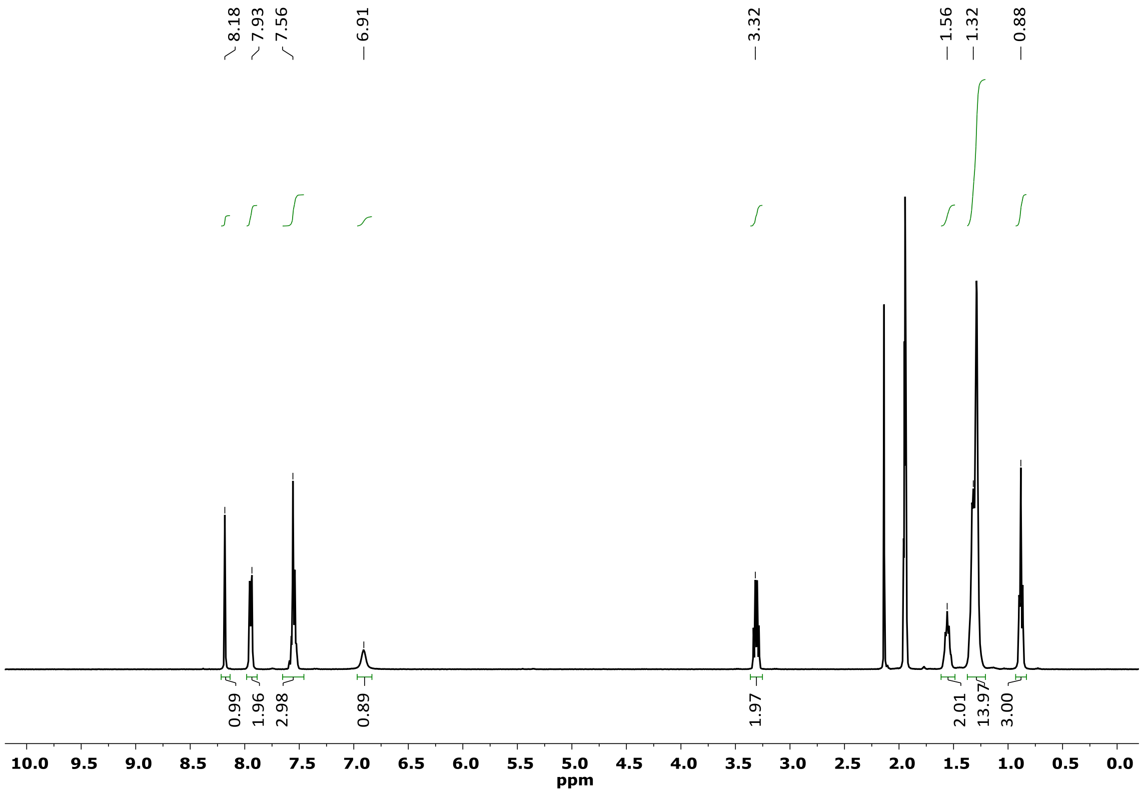

Figure S4 ^{1}H NMR (400 MHz, 301 K, CD_3CN) of **1**.

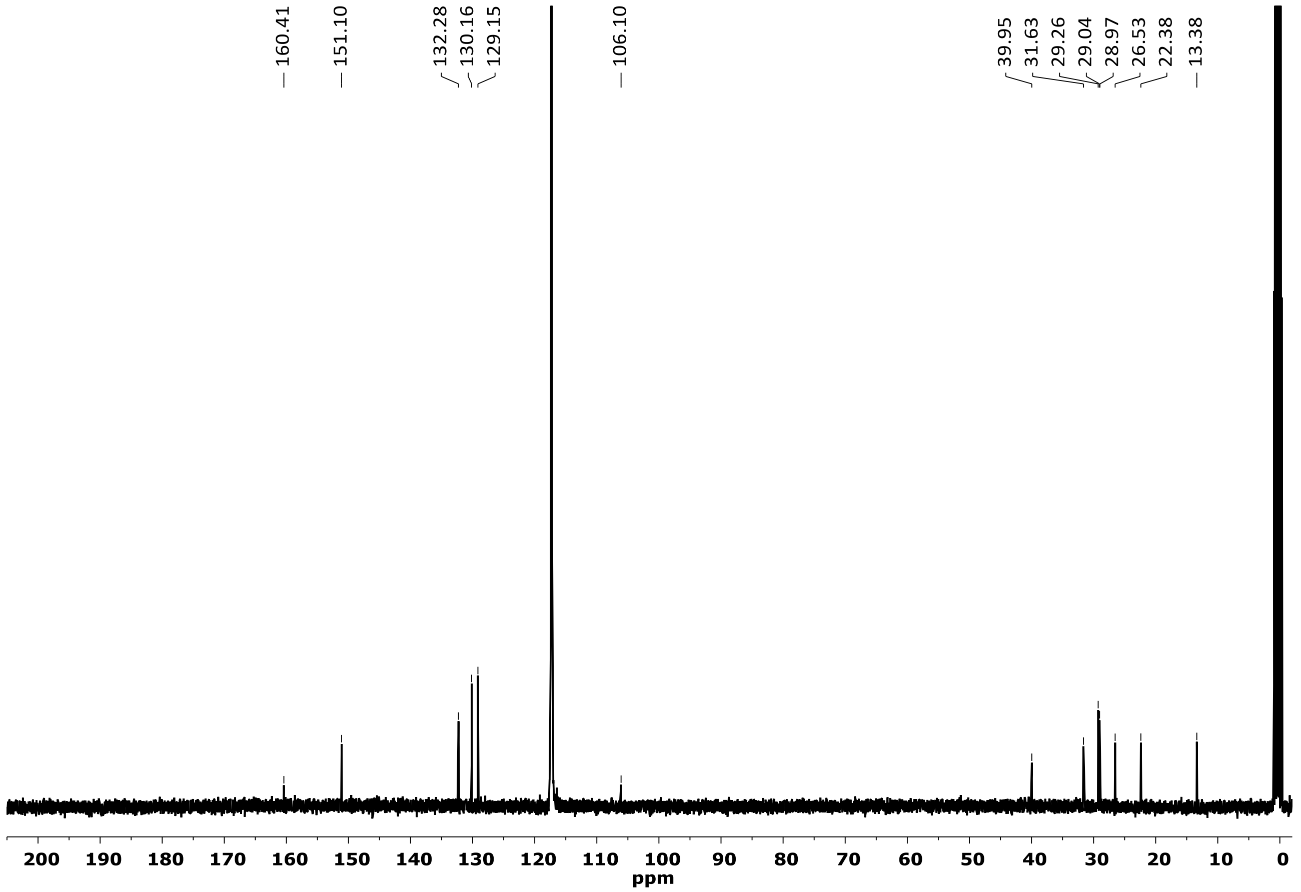

Figure S5 ^{13}C NMR (100 MHz, 301 K, CD_3CN) of **1**.

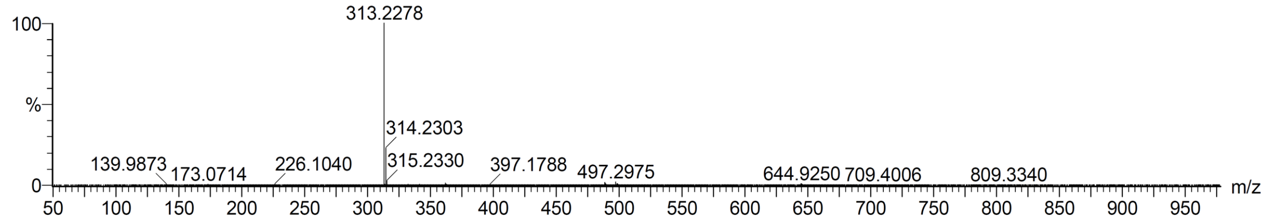

Figure S6 HRMS spectrum of (ESI-TOF) (m/z) of **1**.

3 General procedure for vesicles preparation

Vesicles suspensions were prepared as follows: to a LoB Eppendorf microcentrifuge tubes was added a chloroform solution of lipid 1,2-Dioleoyl-sn-glycero3-phosphocholine (DOPC) and a solution of **1** in methanol. The solvent was removed in vacuo and dried under N_2 for 1 h. Then, the lipids were rehydrated with 50 mM solution of HEPES buffer with NaCl 100 mM at pH 7.2 and sonicated for 1 min. The suspension was subjected to 5 cycles of freeze-thaw using liquid nitrogen. The suspension was extruded 19 times through a polycarbonate filter with 200 nm pores in an Avestin Lipofast apparatus, and then the vesicles were separated by the bulk solution using prepacked GPC columns eluting with the buffer. The vesicles were obtained with a final concentration of 1 mM with a 15% loading of **1** (0.15 mM).

The UV Vis spectra of a vesicles solution (1.0 mM), a solution of **1** in methanol (0.15mM) and a solution of the vesicles with embedded **1** are showed in Figure S1. The spectra show how the incorporation of **1** within the vesicles is confirmed by the presence of an absorption band with maximum absorbance at 300 nm. In addition, LC-MS spectrum of the vesicles solution embedding **1** further confirmed the presence of **1** in the bilayer after the vesicles preparation.

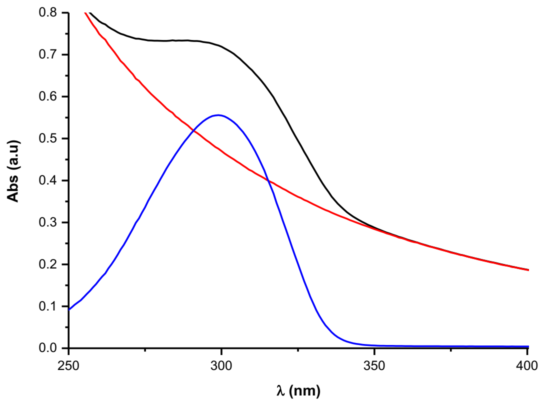

Figure S7 UV-Vis spectra of a solution of vesicles formed by DOPC in HEPES buffer at pH=7.2 (red line, 1 mM), a solution of **1** in methanol (blue line, 0.15 mM) and a solution of **1** (0.15 mM) embedded in DOPC vesicles 1.0 mM in HEPES buffer at pH=7.2 (black line, 0.15 mM).

4 Dynamic Covalent Libraries Analysis

4.1 LC-MS identification of 3a-3d Adducts

To 1.0 ml of a solution 0.15 mM of receptor **1** in methanol were added different aliquots of thiols **2a,2d** from a methanol solution and they were allowed to equilibrate for 1 hour and the reaction was monitored by HPLC-ELSD and LC-MS. In the case of the vesicle systems, receptor **1** (0.15 mM) was embedded in a lipid bilayer (DOPC, 1.0 mM) at pH=7.2 in HEPES buffer. In this case, the thiols were added from a HEPES buffer solution at pH=7.2.

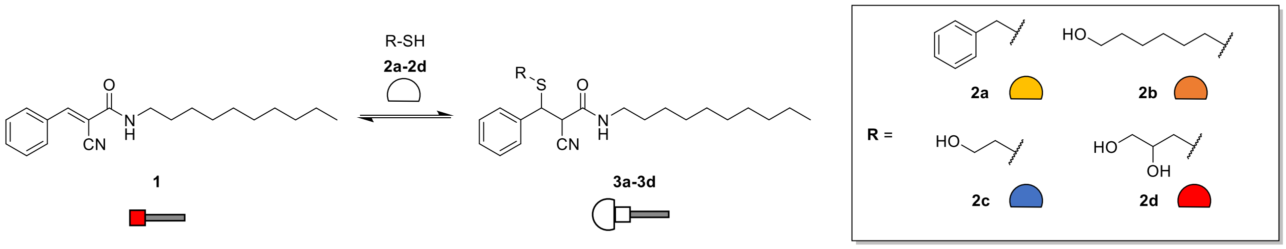

Scheme S1 Addition of thiols **2a-2d** to **1** leads to formation of the corresponding Michael adducts **3a-3d**.

The adducts **3a-3d** formation in the vesicles system and in single phase was monitored by LC-MS and the MS trace with the corresponding MS peaks for each adducts are reported in Figure S8 in the case of vesicles. The adduct formed reported in Figure S8-S9 were obtained adding 1.0 eq of **2a**, 1.8 eq. of **2b,** 12 eq. of **2c** and 15 eq. of **2d.** The same solutions were also analyzed with HPLC-ELSD to allow further quantification in DCL formation experiments (see section S4.2). The adducts formation is reported in Figure S9.

The method employed for the different mixture analysis is reported in the general method paragraph.

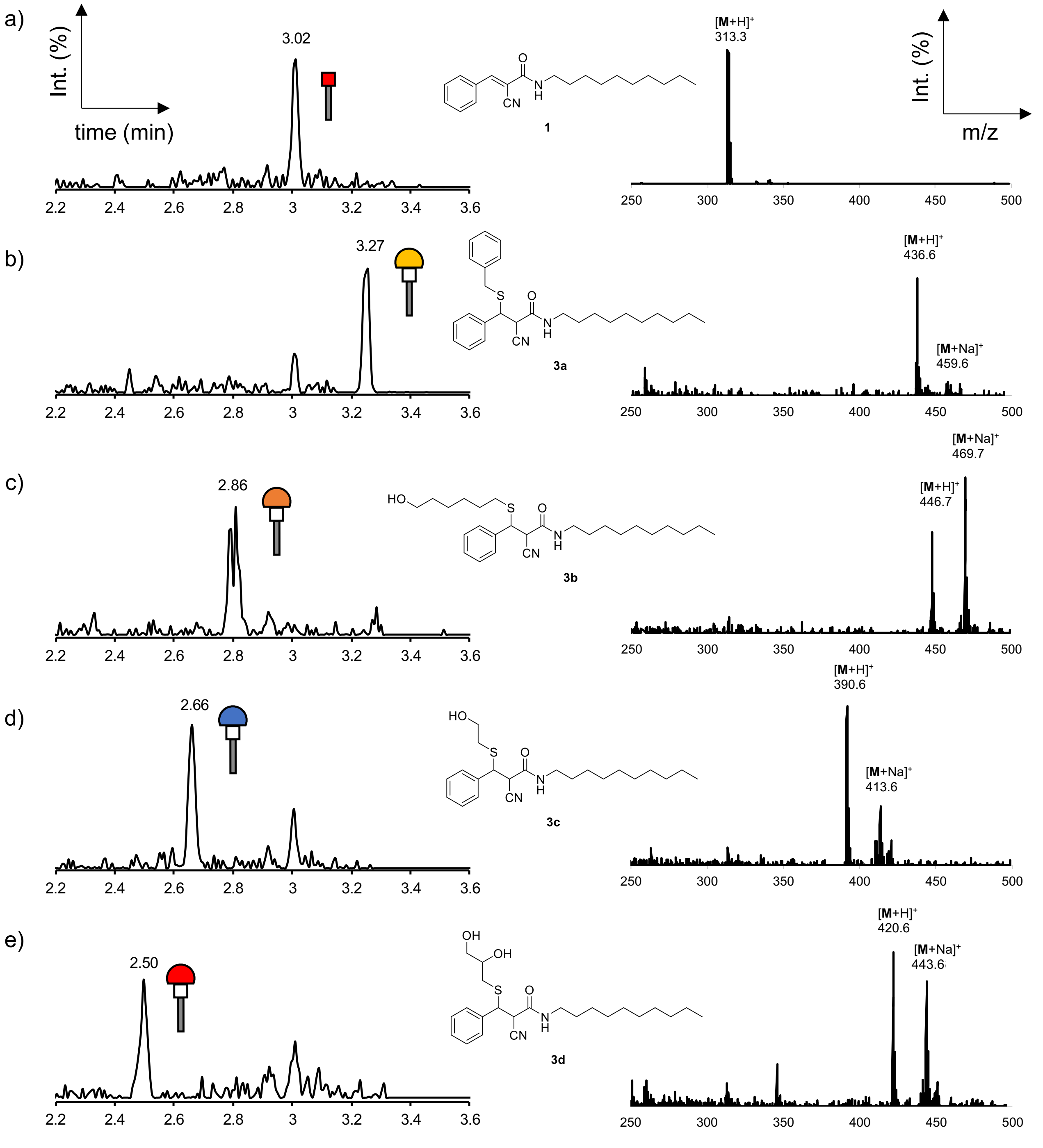

Figure S8 LC-MS traces of compound **1** (a) and the corresponding adducts **3a** (b), **3b** (c), **3c** (d) and **3d** (e) on the left and their mass spectra on the right.

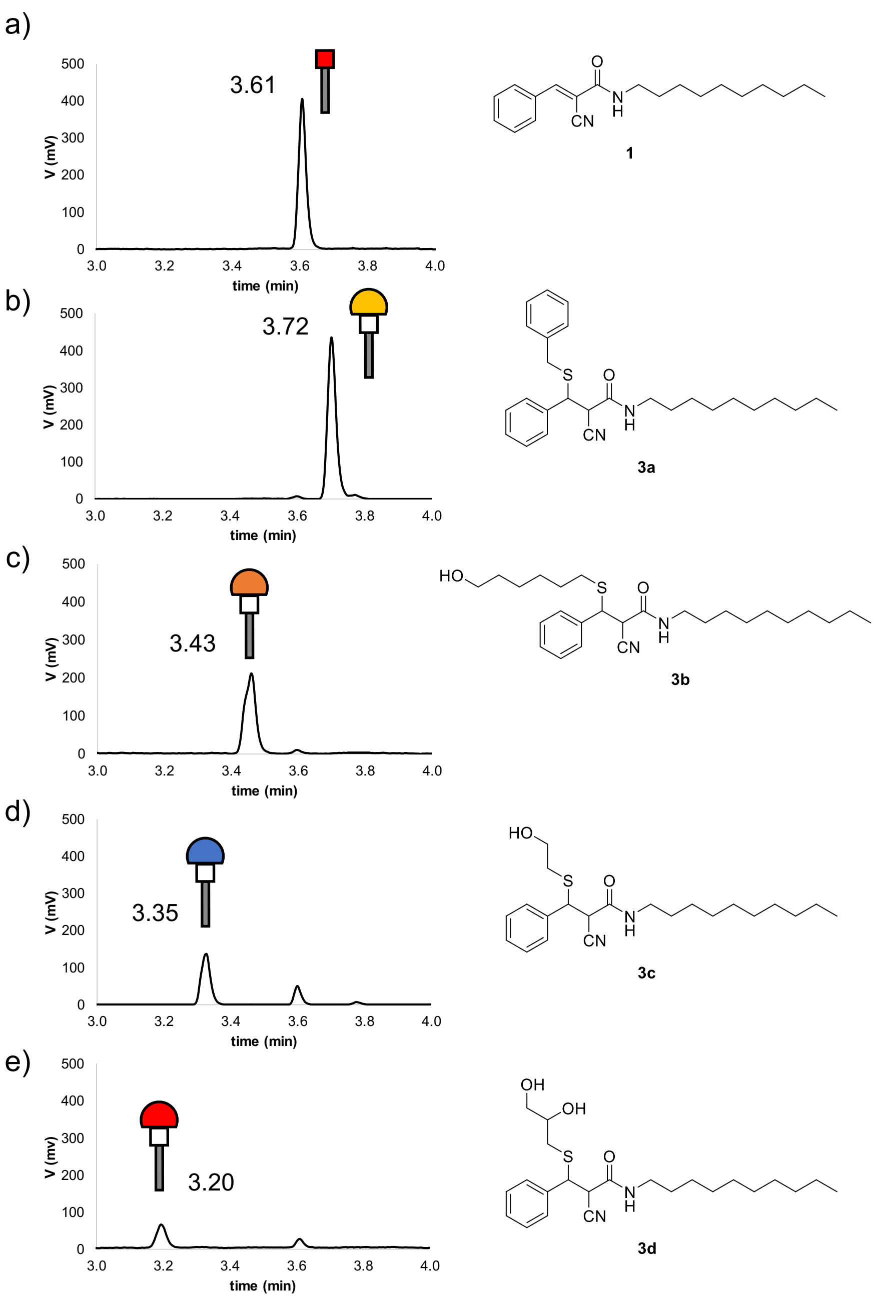

Figure S9 HPLC-ELSD traces of compound **1** (a) and the corresponding adducts **3a** (b), **3b** (c), **3c** (d) and **3d** (e).

4.2 HPLC-ELSD Analysis of the Dynamic Covalent Library

To quantify the formation of the different library components an ELSD detector was employed for the HPLC analysis and the method was developed as follow.

For each library component, it was calculated a detector response factor in methanol. Initially, a series of HPLC-ELSD spectra of compound **1** at known concentration and the corresponding detector response calibration curves of **1** were obtained. The results are showed in Figure S10. The slope obtained by this correlation represents the linear response factor of the detector to **1.**

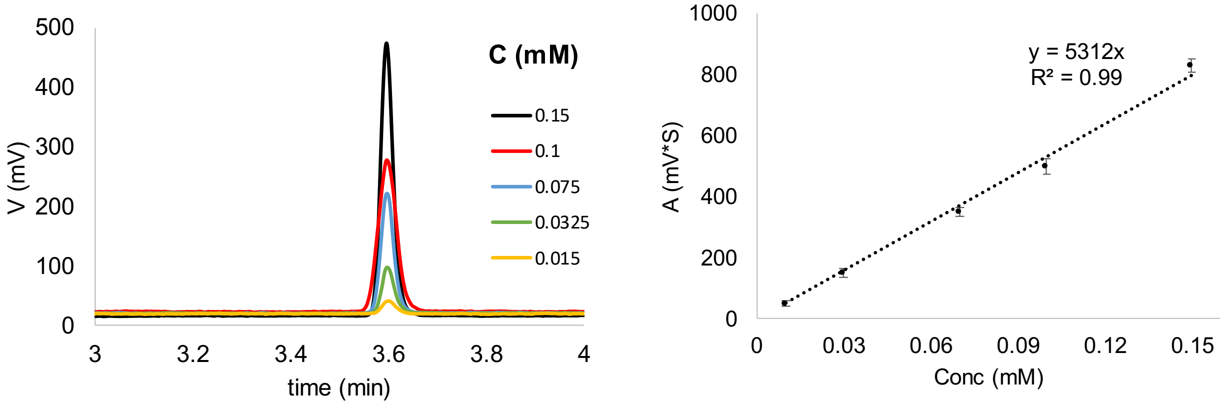

Figure S10 HPLC-ELSD spectra of compound **1** at different concentration (left) and corresponding response factor calibration curve, the response factor is given by the slope (right).

Then, it was possible to obtain the response factor for each adduct formed **3a-3d**. To 1.0 ml of a solution 0.15 mM of receptor **1** in methanol were added different aliquots of thiols **2a,2d** from a methanol solution and they were allowed to equilibrate for 1 hour . The reaction was monitored by HPLC-ELSD.

Since the concentration of **1** is linearly correlating with the ELSD integral area based on the calculated response factor, it was possible to obtain the concentration of each adduct by subtracting the concentration of the unreacted receptor **1** calculated after each thiol addition $[\mathbf{1}]_n$ to the initial concentration of $[\mathbf{1}]_0 = 0.15$ mM (See formula).

$$[\mathbf{3a\text{-}3d}] = [\mathbf{1}]_0 - [\mathbf{1}]_n$$

After that, the response factor for each adduct was calculated by plotting the calculated concentration of the adduct [**3a-3d**] towards the integral area of the adduct formed.

As example the ELSD spectra for the formation of adduct **3b** at different equivalent of thiol **2b** added are shown in Figure S11. The response factor was calculated by plotting the calculated concentration of **3b** towards the integral area of the adduct formed (Figure S11).

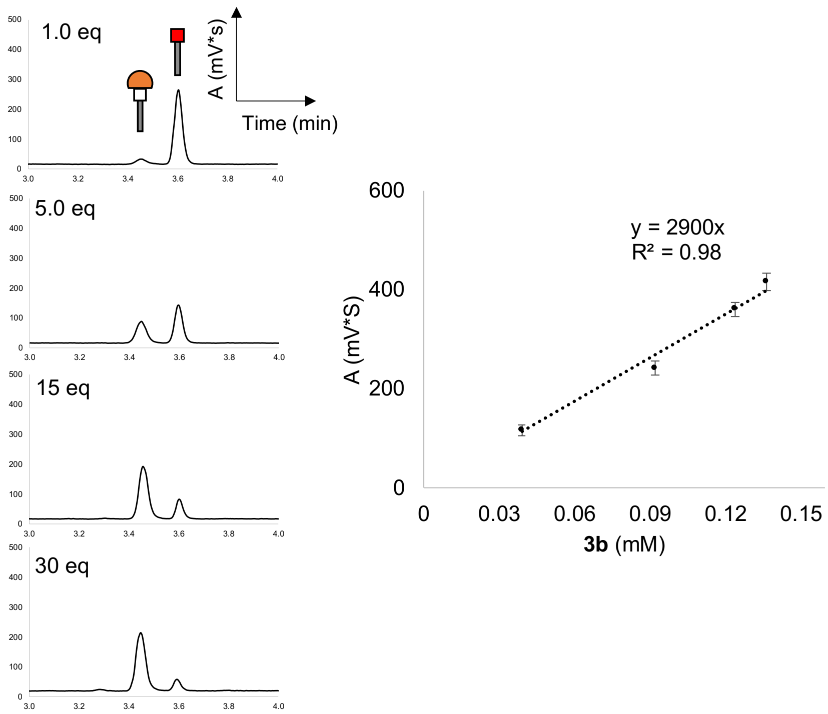

Figure S11 HPLC-ELSD spectra of adduct **3b** formation at different equivalent of thiol **2b** added ([**1**]$_0$=0.15 mM) and corresponding response factor calibration curve, the response factor is given by the slope (right).

Adopting the same approach, the response factors for each adduct were obtained repeating the calibration twice.

From these data, it was possible to obtain the response factors for each adduct which are showed in Table S1 including their characteristic retention time. In addition, the relative response factors used for adducts integral correction are reported in the last column of Table S1 and they were obtained by dividing the response factor of each adduct for the response factor of **1.** Retention times of each adducts **3a-3d** and their relative response factors values were further employed for the identification and quantification of the library components in the complex mixtures in both studied systems.

Compound	Retention time (min)	Response Factor (R.F.) (mV*s*mM^{-1})	(Relative R. F.)$^{-1}$
**1**	3.61	5312 ± 120	1.0
**3a**	3.70	4200 ± 114	1.3
**3b**	3.46	2900 ± 98	1.9
**3c**	3.33	1940 ± 44	2.8
**3d**	3.19	1730 ± 37	3.2

Table S1 Data obtained from ELSD analysis of the integrals and retention peaks.

The inverse of the relative response factor were used in the mixed experiment. By multiplying the integral corresponding to each adduct for the (Relative R. F.)$^{-1}$ we obtained the corrected area of each library component.

Corrected Area (**3a-3d**, **1**) = (Relative R. F.)$^{-1}$ * Experimental Area (**3a-3d**, **1**)

After that, the fraction of each library component was calculated by dividing the corrected area for the total area of the library components.

Fraction % (**3a-3d**, **1**) = (Corrected Area (**3a-3d**, **1**) / ∑ [Corrected Area (**3a-3d**, **1**)])*100

4.3 Dynamic Covalent Library Reversibility Assessment

General Procedure for DCL formation

To 1.0 ml of a solution 0.15 mM of receptor **1** in methanol were added 30 µL of a methanol solution containing thiols **2a**, **2d** (concentration of each thiol 20 mM in the mixture, addition of 1.0 eq. for each thiol) and they were allowed to equilibrate for 1 hour. The final concentration of each thiol in the DCL was 0.15 mM. The reaction was monitored by HPLC-ELSD. In the case of the vesicles systems, receptor **1** (0.15 mM) was embedded in lipid bilayer (DOPC, 1mM) at pH=7.2 in HEPES buffer. In this case, the solution containing all the thiols was prepared in HEPES buffer at pH=7.2.

General Procedure to assess the library reversibility

To 1.0 ml of a solution 0.15 mM of receptor **1** in methanol were added 22.5 µL of a methanol solution containing three thiols of the series **2a**, **2d** (concentration of each thiol in the mixture 20 mM, addition of 1.0 eq. for each thiol) and they were allowed to equilibrate for 1 hour and the reaction was monitored by HPLC-ELSD. After 1 hour 7.5 µL of the last thiol (20 mM) of the series were added and the reaction was monitored until a second equilibration was reached. In the case of vesicles, receptor **1** (0.15 mM) was embedded in a lipid bilayer (DOPC, 1mM) at pH=7.2 in HEPES buffer. In this case, the solution containing all the thiols was prepared in HEPES buffer at pH=7.2.

In Figure S12 is showed a schematic representation for one example. In this case, thiols **2a**, **2b** and **2c** were added to receptor **1** when embedded in the vesicles and the system was allowed to equilibrate for 1 hour (1st step). Then thiol **2a** was added to the system (2nd step). The fractions of the library members observed in this series of experiment are shown for the single phase system (Figure S13) and in the vesicles system (Figure S14).

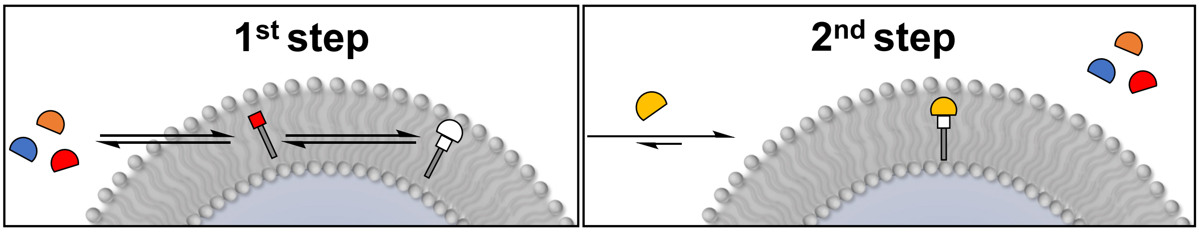

Figure S12. a) Schematic representation of the reversibility experiment. Initially, thiols **2a**, **2b** and **2c** were added to receptor **1** when embedded in the vesicles and the system was allowed to equilibrate for 1 hour (1st step). Then thiol **2a** was added to the system (2nd step).

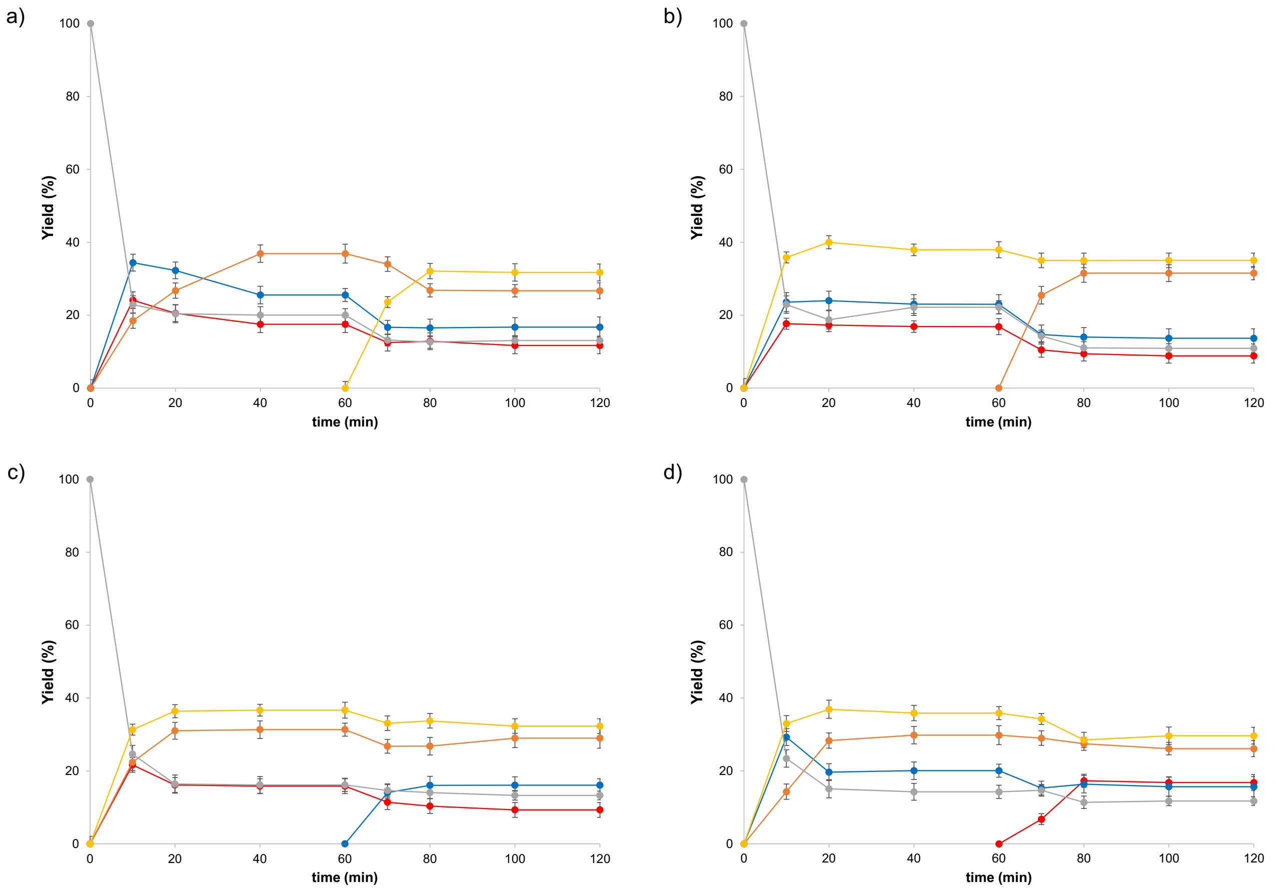

Figure S13 Library members in the single phase system. The system equilibrates in 1 hour toward the formation of adduct and then the last thiol of the series was added. Last thiol added a) **2a**, b) **2b,** c) **2c**, d) **2d**. $[\mathbf{1}]_0$ = 0.15 mM; [**2a**]=[**2b**]=[**2c**]=[**2d**]=[**2e**] = 0.15 mM. [—] = **3a**, [—] = **3b**, [—] = **3c**, [—] = **3d**, [—] = **1**.

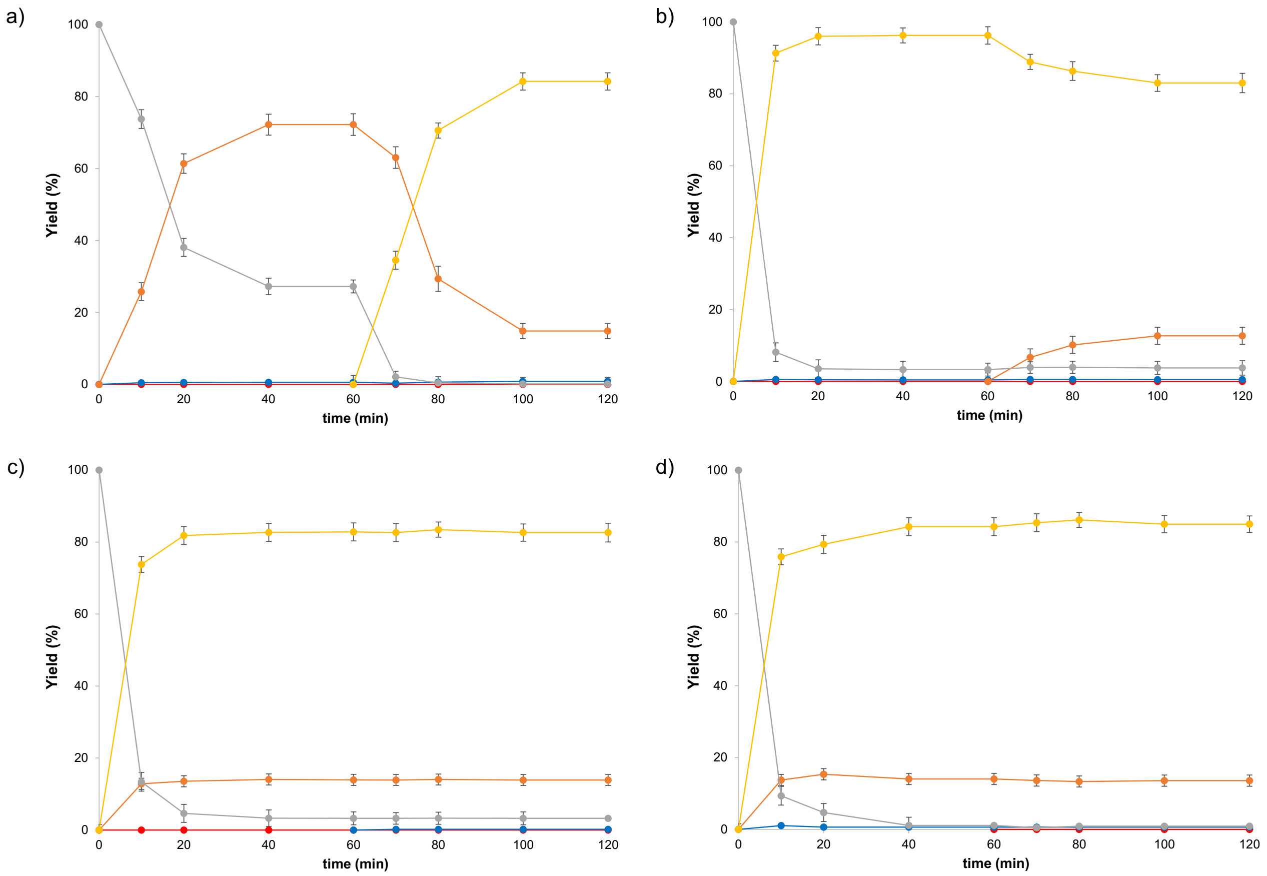

Figure S14 Library members in the vesicle system. The system equilibrates in 1 hour toward the formation of adduct and then the last thiol of the series was added. Last thiol added a) **2a**, b) **2b,** c) **2c**, d) **2d.** [**1**]$_0$ = 0.15 mM; [**2a**]=[**2b**]=[**2c**]=[**2d**]=[**2e**] = 0.15 mM. [—] = **3a**, [—] = **3b**, [—] = **3c**, [—] = **3d**, [—] = **1**.

5 Controlled Product Amplification Triggered by Vesicles

To 1.0 ml of a solution 3.0 mM of receptor **1** in methanol were added 60 μL of a methanol solution containing the thiols of the series **2a,2d** (concentration of each thiol in the mixture 200 mM, addition of 1.0 eq. for each thiol) and they were allowed to equilibrate for 1 hour. The final concentration of each thiol in the DCL was 3.0 mM each. The reaction was monitored by HPLC-ELSD. In Figure S15a the spectra at different equilibration times are showed.

After 60 minutes, an aliquot of the solution containing the DCL (100 μL) was injected into 2.0 ml of a 1.0 mM DOPC vesicles solution in HEPES buffer at pH=7.2, achieving a final concentration of the system of 0.15 mM for each component of the library. The HPLC-ELSD spectra at different equilibration time are reported in Figure S15b.

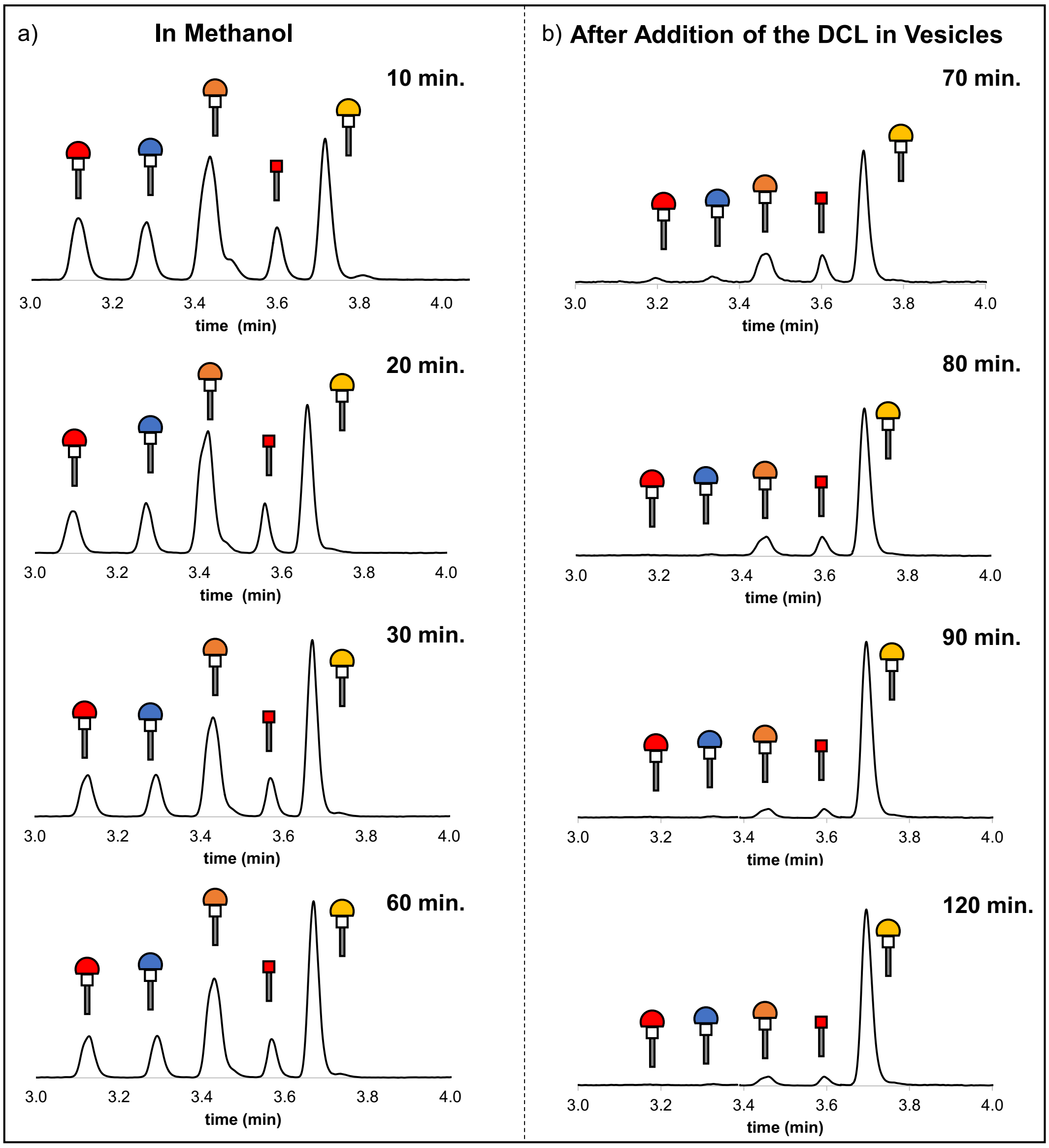

Figure S15 HPLC ELSD spectra of the library members in methanol (a) and after the addition of an aliquot of the DCL in vesicles (b). The fraction of each product was calculated from the integral of HPLC trace corrected by the relevant response factor. These spectra are referred to Figure 3 in the manuscript.

As a control, the same experiment was performed in the absence of vesicles by adding an aliquot of the methanol solution containing the DCL (100 µL) into 2.0 ml of HEPES buffer aqueous solution at pH=7.2, achieving a final concentration of the system of 0.15 mM for each component of the library. The HPLC-ELSD spectra after the equilibration are reported in Figure S16b (the corresponding results obtained in methanol are shown in Figure S16a). The time course of equilibration in aqueous buffer with no vesicles present is reported in Figure S16c.

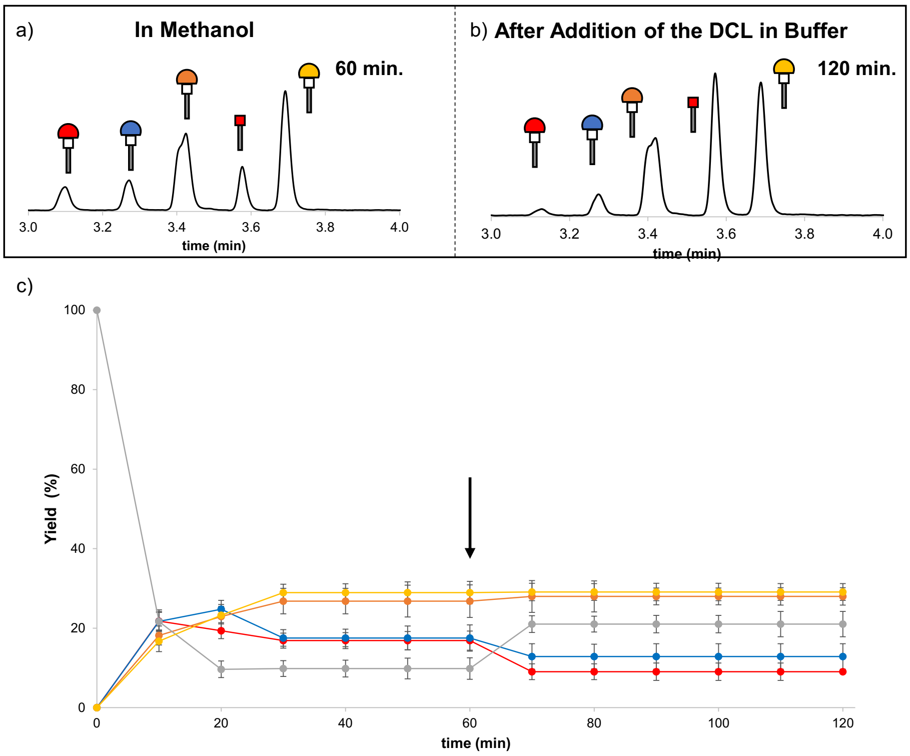

Figure S16. HPLC traces of the product distribution obtained 1 hour after mixing **1** (3.0 mM) with **2a-2e** (3.0 mM each) (a) in methanol, and (b) after addition of this mixture to a HEPES buffer at pH 7.2 aqueous solution. c) Time course for equilibration of the dynamic covalent library. At time zero, **1** (3 mM) was mixed with **2a-2e** (3 mM each) in 1.0 mL of methanol. After 1 hour (arrow), an aliquot of this mixture (0.1 ml) was added to 2.0 mL of a HEPES buffer at pH 7.2 aqueous solution. The fraction of each product was calculated from the integrals of the HPLC traces corrected by the relevant response factors. [—] = **3a**, [—] = **3b**, [—] = **3c**, [—] = **3d**, [—] = **1**.

6 Kinetic Experiments and Binding Constant Determination

6.1 UV-Vis spectra of thiol addition in vesicles

Kinetics of thiols **2a-2d** addition to conjugate acceptor **1** embedded in vesicles were monitored with UV-Vis spectroscopy. To vesicles formed by DOPC (1mM, HEPES buffer, pH=7.2) loaded with receptor **1** (15% loading, 0.15 mM) were added different equivalent of thiols **2a-2d** in buffer from stock solutions at 20 mM concentration. UV Vis spectra of kinetic experiments related to the addition of 1.0 eq. of each thiol **2a-2d** to the system is reported in Figure S17.

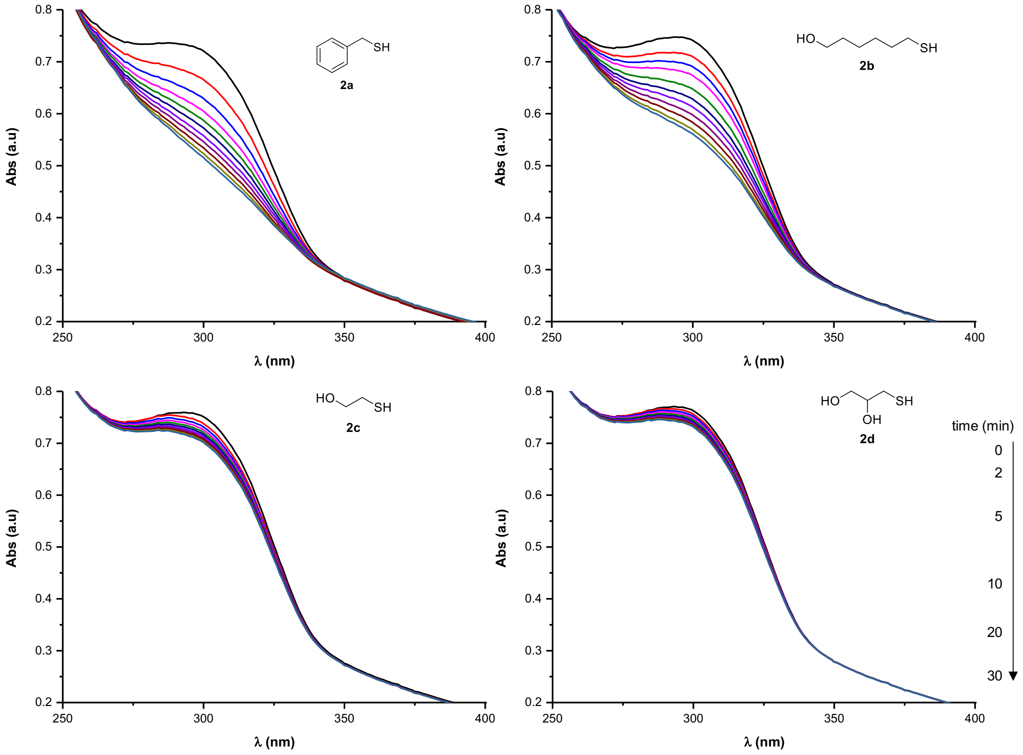

Figure S17 Selected UV-Vis spectra of adduct **3a-3d** formation. To the vesicles loaded with **1** (black line) (0.15 mM) were added 1.0 eq. (0.15 mM) of different thiols **2a-2d**.

6.2 UV-Vis spectra of thiol addition in methanol

Kinetics of thiols **2a-2d** addition to conjugate acceptor **1** in methanol solution were monitored with UV-Vis spectroscopy. To **1** (0.15 mM) were added different equivalent of thiols **2a-2d** from a 60 mM methanol solution. UV Vis spectra of kinetic experiments related to the addition of 10 eq. of each thiol **2a-2d** to the system is reported in Figure S18.

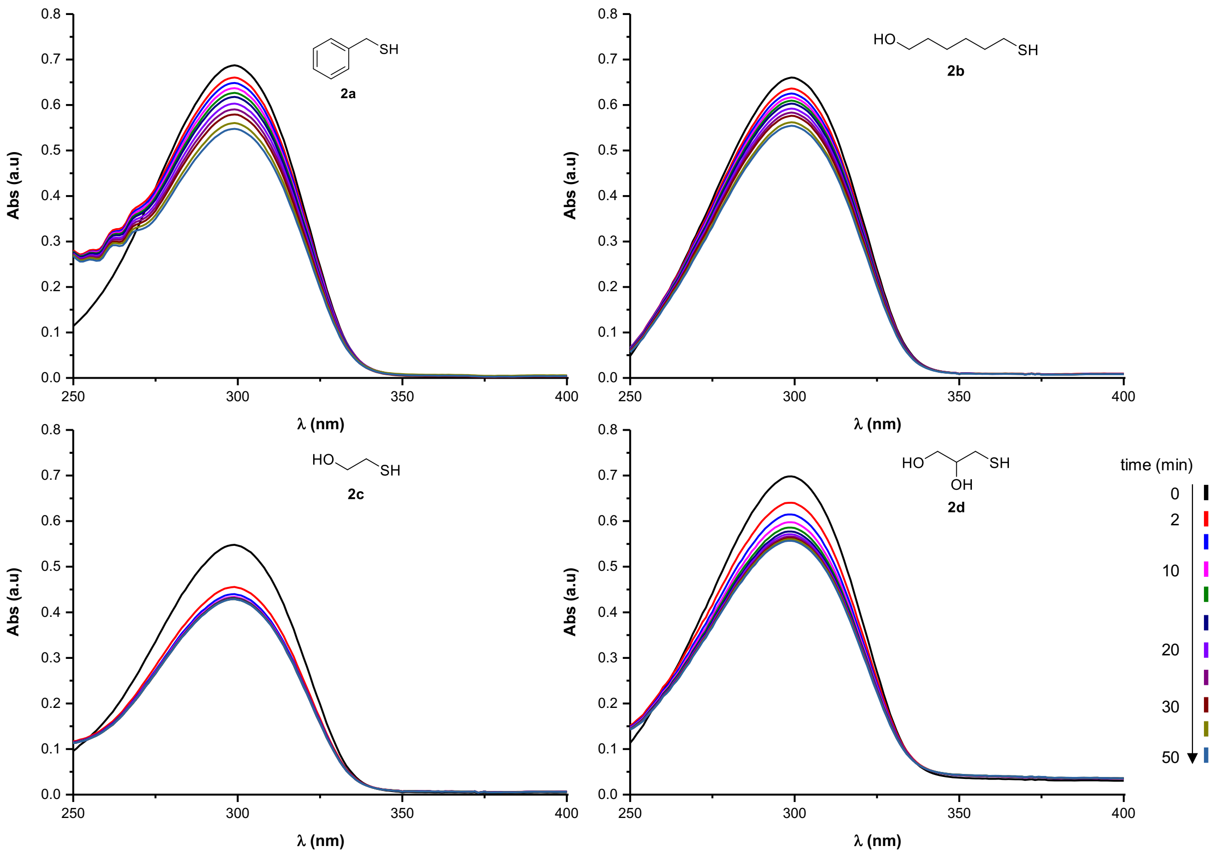

Figure S18 Selected UV-Vis spectra of adduct **3a-3d** formation. To a methanol solution of **1** (0.15 mM, black line) were added 10 eq. (1.5 mM) of different thiols **2a-2d**.

6.3 Kinetic profile of the adduct formation and binding constant determination

The formation of the adducts was monitored taking into account that the absorbance of **1** at 300 nm is quenched after addition of thiols,[1] therefore it was possible to calculate the concentration of adduct **3a-3d** formed at the equilibrium considering the formula

$$[\boldsymbol{Adduct}] = [\mathbf{1}]_{\mathbf{0}} * \frac{[A_0 - A_t]}{[A_0 - A_{ves}]}$$

In which $[\mathbf{1}]_{\mathbf{0}}$ is the initial concentration of the receptor embedded in the bilayer (0.15 mM), A_0 is the absorbance at 300 nm of the system before the thiol addition, A_t is the absorbance of the system at 300 nm at the time when the equilibrium is reached, and A_{ves} is the absorbance estimated from a linear fitting of UV-Vis spectra which consider only the absorbance of the vesicles solution at 300 nm. In the case of methanol, the term A_{ves} becomes 0 due to the absence of absorbance of the adducts at 300 nm.

The kinetic trend for the formation of adducts **3a-3d** at different equivalents of thiols added respect to **1** are reported using vesicles (Figure S19-S22) and in methanol solution (Figure S23-S26). After the determination of adducts concentration at the equilibrium, it was possible to obtain the association constants (*K*) for the reversible adducts formation since the concentration in solution of all the species was known. *K* was calculated using the following formula

$$K = \frac{[\boldsymbol{Adduct}]}{[\mathbf{1}] * [\boldsymbol{thiol}]}$$

In which the concentration of the adduct was calculated taking into account of the above mentioned formula, [**1**] is the concentration of the receptor at the equilibrium corresponding to ($[\mathbf{1}]_0$ – [**Adduct**]) and [**thiol**] is the concentration of free thiol at the equilibrium given by ($[\mathbf{thiol}]_0$ – [**Adduct**]). The same approach was adopted for the binding constant determination in methanol for the formation of **3a-3d**.

In Table S2-S5 are reported the concentration values for each species at the equilibrium when the adducts were formed in vesicles. In Table S6-S9 are reported the concentration values when the adducts were formed in methanol. In the last column of each table are reported the binding constant calculated at each equilibration point. The average binding constant value for each system is given with the related error and they are reported in Table S10 for both systems.

Vesicles

[**2a**]$_0$ / [**1**]$_0$	[**2a**]$_0$ (mM)	[**3a**] (mM)	[**1**] (mM)	[**2a**] (mM)	***K*** (1*10^5 M^{-1})
0.2	0.030	0.029	0.123	0.001	1.79
0.4	0.060	0.057	0.093	0.003	2.04
0.6	0.090	0.083	0.057	0.007	2.08
0.8	0.120	0.106	0.034	0.014	2.23
1.0	0.150	0.121	0.019	0.029	2.20

Table S2 Concentration value of each species at the equilibrium reported for each addition of thiol **2a** to **1** in vesicles.

[**2b**]$_0$ / [**1**]$_0$	[**2b**]$_0$ (mM)	[**3b**] (mM)	[**1**] (mM)	[**2b**] (mM)	***K*** (1*10^4 M^{-1})
0.2	0.030	0.024	0.126	0.006	3.17
0.4	0.060	0.044	0.106	0.016	2.59
0.6	0.090	0.062	0.078	0.028	2.84
1.0	0.150	0.088	0.052	0.062	2.73
1.8	0.270	0.114	0.026	0.156	2.81

Table S3 Concentration value of each species at the equilibrium reported for each addition of thiol **2b** to **1** in vesicles.

[**2c**]$_0$ / [**1**]$_0$	[**2c**]$_0$ (mM)	[**3c**] (mM)	[**1**] (mM)	[**2c**] (mM)	***K*** (M^{-1})
1.0	0.150	0.024	0.126	0.126	1500
2.0	0.300	0.042	0.108	0.258	1510
5.3	0.800	0.078	0.062	0.722	1740
8.0	1.200	0.092	0.048	1.108	1730
12.0	1.800	0.105	0.035	1.695	1770

Table S4 Concentration value of each species at the equilibrium reported for each addition of thiol **2c** to **1** in vesicles.

[**2d**]$_0$ / [**1**]$_0$	[**2d**]$_0$ (mM)	[**3d**] (mM)	[**1**] (mM)	[**2d**] (mM)	***K*** (M^{-1})
1.0	0.150	0.016	0.134	0.134	890
4.0	0.600	0.046	0.104	0.554	800
8.0	1.200	0.065	0.075	1.135	760
12.0	1.800	0.084	0.056	1.716	870
14.7	2.200	0.095	0.045	2.105	1000

Table S5 Concentration value of each species at the equilibrium reported for each addition of thiol **2d** to **1** in vesicles.

Methanol

[**2a**]$_0$ / [**1**]$_0$	[**2a**]$_0$ (mM)	[**3a**] (mM)	[**1**] (mM)	[**2a**] (mM)	***K*** (M^{-1})
1.7	0.25	0.025	0.125	0.225	870
3.3	0.49	0.037	0.113	0.453	720
6.4	0.96	0.062	0.078	0.898	880
10.0	1.50	0.072	0.068	1.428	740
15.3	2.30	0.082	0.048	2.218	760

Table S6 Concentration value of each species at the equilibrium reported for each addition of thiol **2a** to **1** in methanol.

[**2b**]$_0$ / [**1**]$_0$	[**2b**]$_0$ (mM)	[**3b**] (mM)	[**1**] (mM)	[**2b**] (mM)	***K*** (M^{-1})
1.7	0.25	0.020	0.130	0.230	680
3.3	0.49	0.032	0.118	0.458	590
6.4	0.96	0.048	0.092	0.912	570
10.0	1.50	0.064	0.076	1.436	590
15.3	2.30	0.073	0.057	2.227	570

Table S7 Concentration value of each species at the equilibrium reported for each addition of thiol **2b** to **1** in methanol.

[**2c**]$_0$ / [**1**]$_0$	[**2c**]$_0$ (mM)	[**3c**] (mM)	[**1**] (mM)	[**2c**] (mM)	***K*** (M^{-1})
1.7	0.25	0.011	0.139	0.239	330
3.3	0.49	0.018	0.132	0.472	290
6.4	0.96	0.032	0.108	0.928	320
10.0	1.50	0.045	0.095	1.455	330
15.3	2.30	0.055	0.075	2.245	330

Table S8 Concentration value of each species at the equilibrium reported for each addition of thiol **2c** to **1** in methanol.

[**2d**]$_0$ / [**1**]$_0$	[**2d**]$_0$ (mM)	[**3d**] (mM)	[**1**] (mM)	[**2d**] (mM)	***K*** (M^{-1})
6.4	0.96	0.025	0.125	0.935	210
10.0	1.50	0.038	0.112	1.462	230
15.3	2.30	0.042	0.098	2.258	190
18.7	2.80	0.055	0.085	2.745	230
20.0	3.00	0.061	0.069	2.939	300

Table S9 Concentration value of each species at the equilibrium reported for each addition of thiol **2d** to **1** in methanol.

**Adduct**	***K* Vesicles (M^{-1})**	***K* Methanol (M^{-1})**
**3a**	(2.07 ± 0.17)*10^5	800 ± 70
**3b**	(2.83 ± 0.22)*10^4	600 ± 50
**3c**	1650 ± 130	320 ± 20
**3d**	860 ± 90	230 ± 40

Table S10 Binding constant values obtained for the formation of adducts **3a-3d** in vesicles and in methanol.

Vesicles

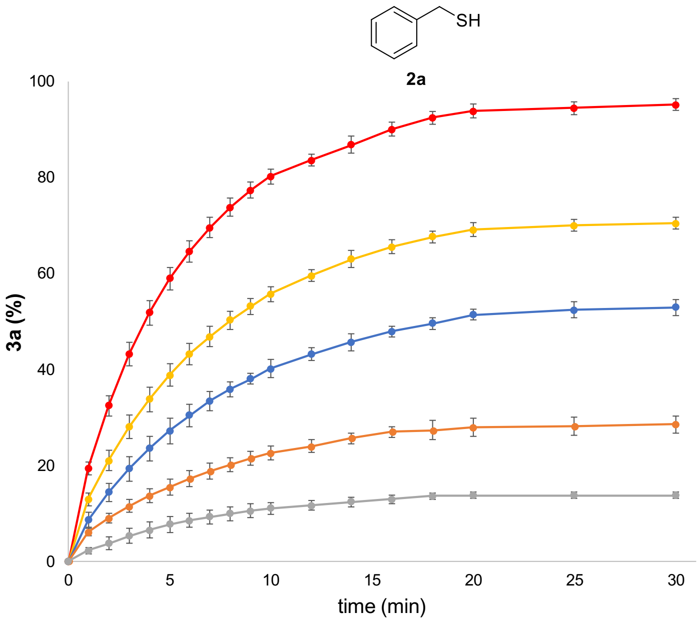

Figure S19 Kinetic trend for the formation of adducts **3a** within vesicles after the addition of thiol **2a**: — 1.0 eq.; — 0.8 eq.; — 0.6 eq.; — 0.4 eq.; — 0.2 eq. [**1**] =0.15 mM

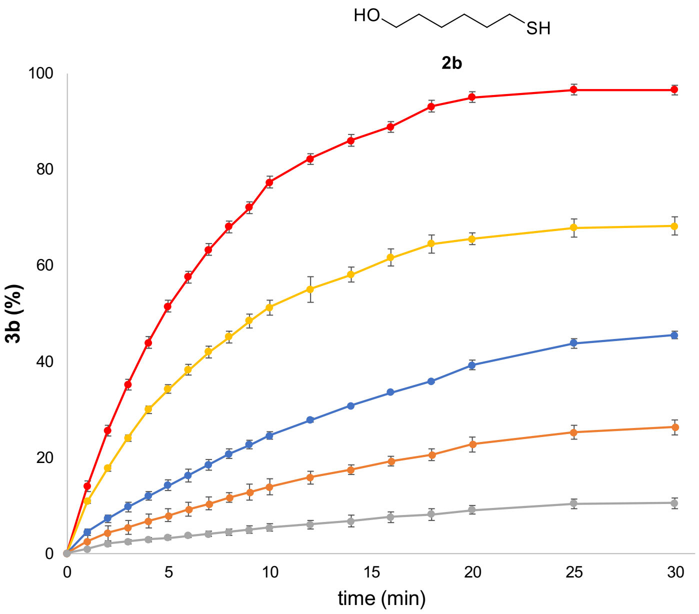

Figure S20 Kinetic trend for the formation of adducts **3b** within vesicles after the addition of thiol **2b**: — 1.8 eq.; — 1.0 eq.; — 0.6 eq.; — 0.4 eq.; — 0.2 eq. [**1**]$_0$=0.15 mM.

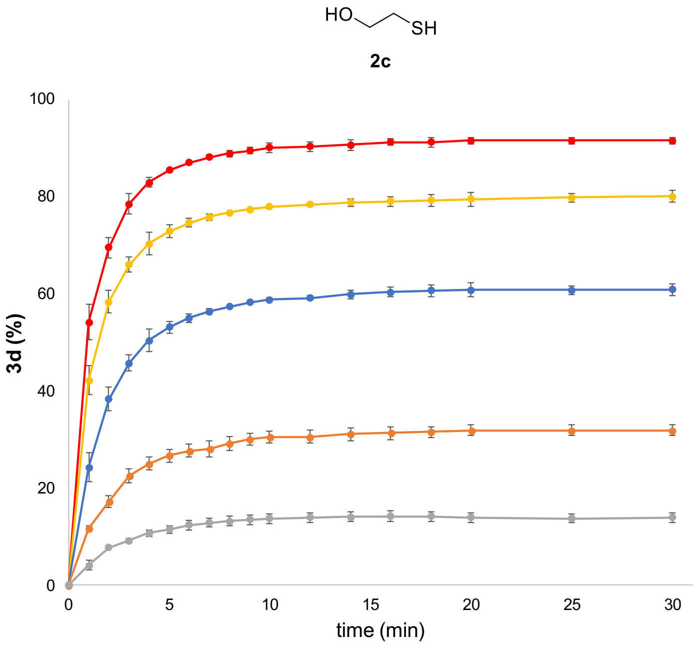

Figure S21 Kinetic trend for the formation of adducts **3d** within vesicles after the addition of thiol **2d**: — 12 eq.; — 8.0 eq.; — 5.0 eq.; — 2.0 eq.; — 1.0 eq. [**1**]$_0$ =0.15 mM.

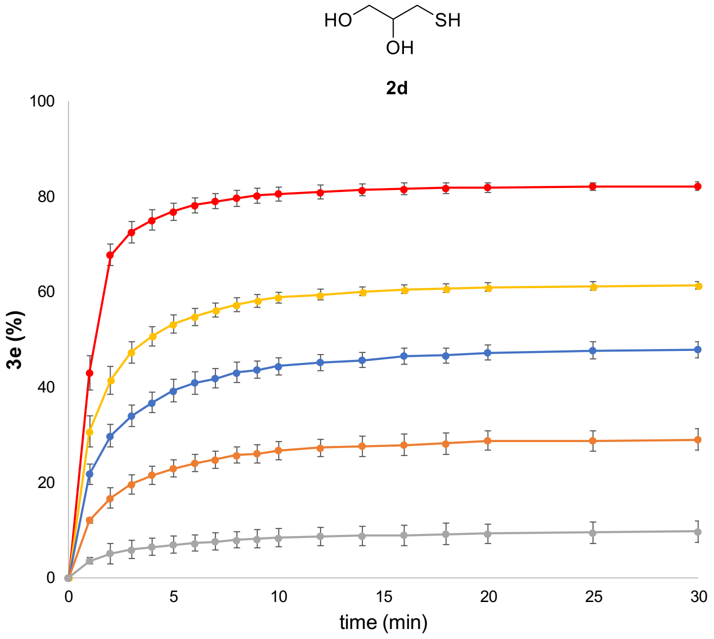

Figure S22 Kinetic trend for the formation of adducts **3d** within vesicles after the addition of thiol **2d**: — 15 eq.; — 12 eq.; — 8.0 eq.; — 4.0 eq.; — 1.0 eq. [**1**]$_0$=0.15 mM.

Methanol

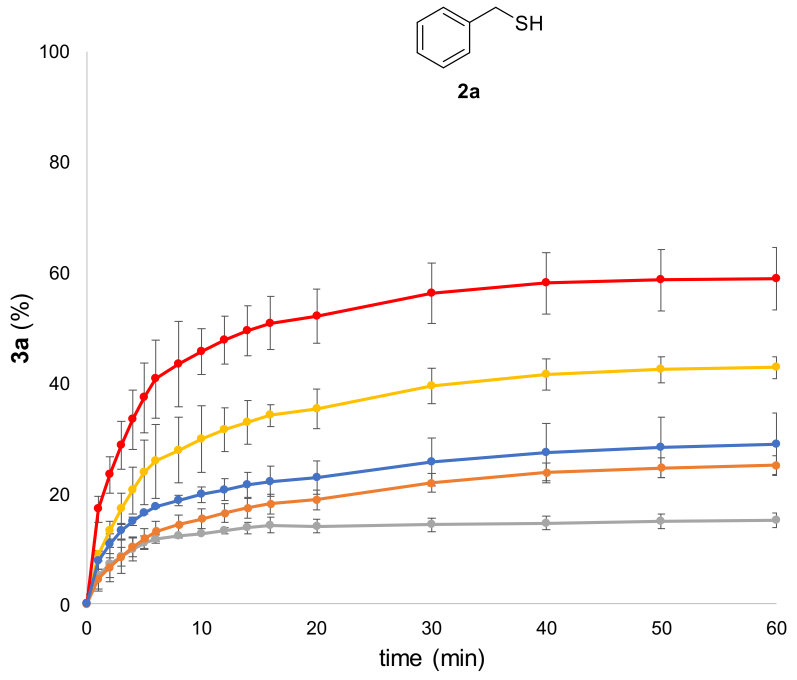

Figure S23 Kinetic trend for the formation of adducts **3a** in methanol after the addition of thiol **2a**: — 15 eq.; — 10 eq.; — 6.4 eq.; — 3.3 eq.; — 1.7 eq. [**1**]$_0$=0.15 mM.

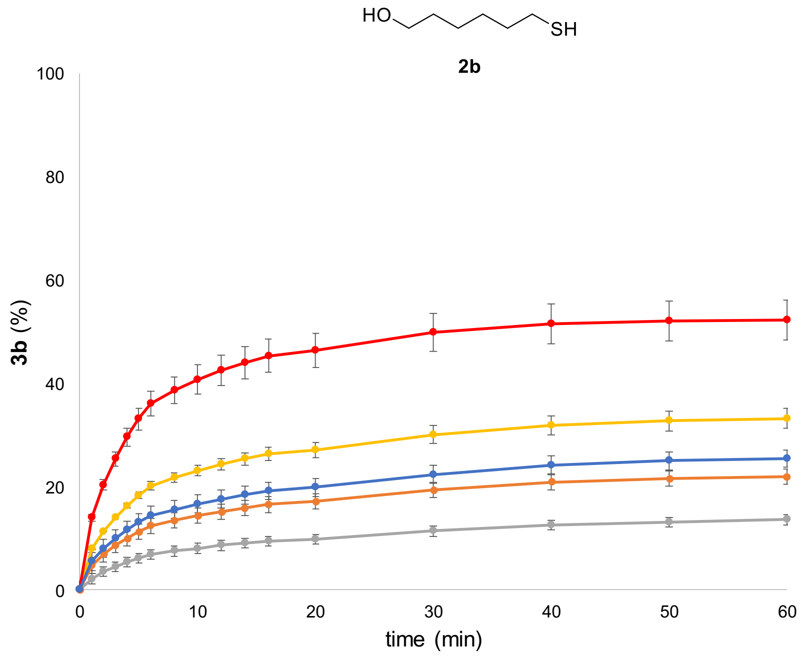

Figure S24 Kinetic trend for the formation of adducts **3b** in methanol after the addition of thiol **2b**: — 15 eq.; — 10 eq.; — 6.4 eq.; — 3.3 eq.; — 1.7 eq. [**1**]$_0$=0.15 mM.

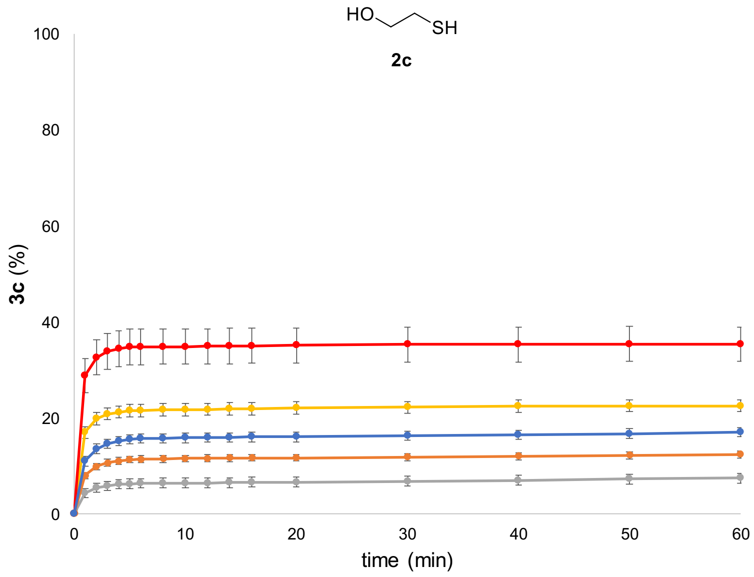

Figure S25 Kinetic trend for the formation of adducts **3c** in methanol after the addition of thiol **2c**: — 15 eq.; — 10 eq.; — 6.4 eq.; — 3.3 eq.; — 1.7 eq. [**1**]$_0$=0.15 mM.

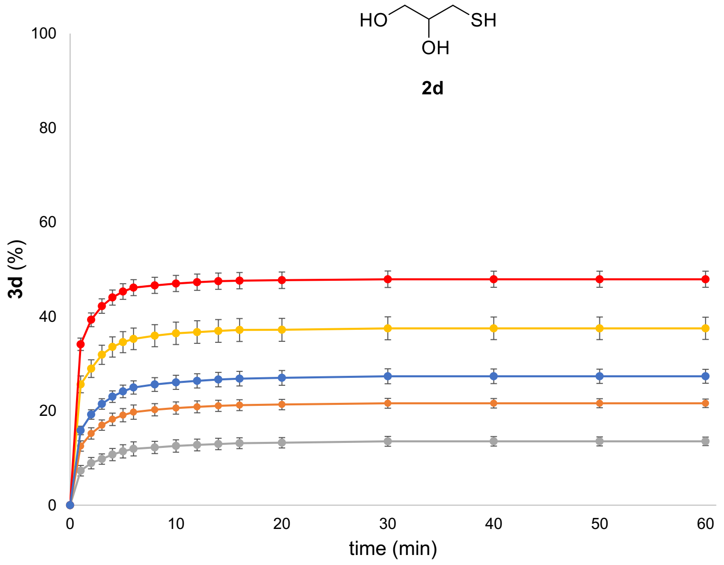

Figure S26 Kinetic trend for the formation of adducts **3d** in methanol after the addition of thiol **2d**: — 20 eq.; — 18 eq.; — 15 eq.; — 10 eq.; — 6.4 eq. [**1**] =0.15 mM.

References

1 I. M. Serafimova, M. A. Pufall, S. Krishnan, K. Duda, M. S. Cohen, R. L. Maglathlin, J. M. McFarland, R. M. Miller, M. Frödin and J. Taunton, *Nat. Chem. Biol.*, 2012, **8**, 471–476.
